# Accelerated evolution of a minimal 63–amino acid dual transcription factor

**DOI:** 10.1126/sciadv.aba2728

**Published:** 2020-06-10

**Authors:** Andreas K. Brödel, Rui Rodrigues, Alfonso Jaramillo, Mark Isalan

**Affiliations:** 1 Department of Life Sciences, Imperial College London, London SW7 2AZ, UK.; 2 Warwick Integrative Synthetic Biology Centre and School of Life Sciences, University of Warwick, Coventry CV4 7AL, UK.; 3 CNRS-UMR8030, Laboratoire iSSB and Université Paris-Saclay and Université d’Évry and CEA, DRF, IG, Genoscope, Évry 91000, France.; 4 Institute for Integrative Systems Biology (I2SysBio), University of Valencia-CSIC, 46980 Paterna, Spain.

## Abstract

Transcription factors control gene expression in all life. This raises the question of what is the smallest protein that can support such activity. In nature, Cro from bacteriophage λ is one of the smallest known repressors (66 amino acids), and activators are typically much larger (e.g., λ cI, 237 amino acids). Previous efforts to engineer a minimal activator from λ Cro resulted in no activity in vivo in cells. In this study, we show that directed evolution results in a new Cro activator-repressor that functions as efficiently as λ cI in vivo. To achieve this, we develop phagemid-assisted continuous evolution (PACEmid). We find that a peptide as small as 63 amino acids functions efficiently as an activator and/or repressor. To our knowledge, this is the smallest protein activator that enables polymerase recruitment, highlighting the capacity of transcription factors to evolve from very short peptide sequences.

## Teaser

The smallest gene activator-repressor with polymerase recruitment is engineered using a 63–amino acid peptide.

## INTRODUCTION

DNA binding proteins that regulate transcription initiation and control gene expression are called transcription factors (TFs). The question of, “What is the smallest peptide that can function as a TF?” is a fundamental one, with broad implications for the evolution of gene regulation (*1*–*3*). However, the potential for a peptide to be a minimal TF depends on its function: whether it is an activator, a repressor, or has dual activity (table S1).

In bacteria, TFs usually recruit or block RNA polymerase to activate or repress genes of interest, respectively. Generally, repression is more straightforward to achieve because it only requires a DNA binding protein to occlude key motifs in a promoter or to block transcription elongation by “roadblock” (*4*). By contrast, activator TFs have to strike a balance between DNA binding, RNA polymerase recruitment, and RNA polymerase release to initiate transcription efficiently (*5*). Consequently, activators and dual TFs might need to be larger and more complex than repressors (table S1). These include well-studied examples such as λ cI (237 amino acids) from bacteriophage λ’s genetic switch (fig. S1A) (*6*).

In this study, we set out to test the minimal size limits for TFs, and in particular whether activators or dual TFs could be made as small as the smallest known repressors. Viruses have some of the smallest functional TFs, and phage λ Cro protein is one of the smallest TF repressors characterized to date, containing only 66 amino acids (*7*, *8*). Cro controls the viral life cycle along with λ cI, and together, they function naturally as a toggle switch (fig. S1B) (*6*). Other examples of very small repressors are Cro from *Enterobacteria* phage P22 (61 amino acids) (*9*) and CopG (45 amino acids) (*10*).

It has been previously shown that Cro might potentially be converted into an activator by transferring the activating surface patch of λ cI onto the surface of Cro (*11*). This can be done as molecular models of λ cI and Cro suggest that α helices two and three of each protein lie in nearly identical positions. As a result, α helices of both TFs are positioned to interact with the same part of RNA polymerase, and Cro might become an activator if α helix two were suitably engineered. However, a rationally engineered Cro variant had only a trace of detectable activity in vitro and none in vivo, in *Escherichia coli*, because of its low affinity for λ operators (*11*). Cro is significantly smaller than common TFs [e.g., LacI, 360 amino acids; TetR, 207 amino acids (table S1)], making it an ideal scaffold for developing a new set of small or minimal activators or dual TFs (fig. S1B). In addition, a set of Cro activators would complement the λ cI synthetic biology toolbox (*12*) for gene circuit engineering in bacterial cells (*13*, *14*), because cI and Cro function on related operators.

## RESULTS

### M13 phagemid-assisted continuous evolution

**Fig. 1:**
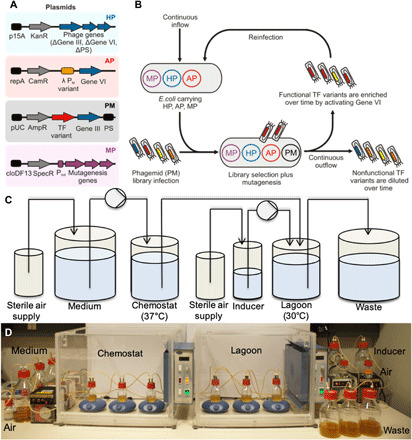
PACEmid of TFs. (**A**) Plasmids: HP, helper phage to provide all phage genes except for gIII and gVI; AP, AP to provide conditional Gene VI expression to enable selection of a successful evolving TF variant; PM, phagemid containing an evolving TF variant and gIII; MP, A chemically inducible mutagenesis plasmid (MP) can, optionally, be used in addition to combinatorial library selection. (**B**) Continuous selection flow diagram: Host cells carrying the HP, AP, and, optionally, an MP continuously flow from a chemostat into a lagoon where they get infected with M13 phage. Only an active TF induces Gene VI expression to complete the phage life cycle, thus enriching this library variant; nonfunctional TF variants are diluted over time. Diversification of the target gene is obtained by combinatorial libraries and/or random mutagenesis. (**C**) Flow chart of the PACEmid continuous evolution system. *E. coli* cells (containing HP, AP, and MP) are cultured in the late log phase (chemostat, 37°C) and flow through a lagoon (30°C) containing the evolving phagemid (PM). (**D**) Photo of bioreactor setup showing three independent experiments performed in parallel (Photo credit: M. Mielcarek, Imperial College London).

To obtain a set of small transcriptional activators derived from λ Cro, we converted our phagemid-based batch selection system (*12*, *15*) for accelerated continuous evolution in a manner similar to phage-assisted continuous evolution (PACE) (*16*, *17*). One difference between our system and classic PACE is that we use phagemids rather than phage to facilitate screening large libraries. The smaller size of the phagemid enables the construction of gene libraries with a much higher number of variants than standard phage. We also prevent bystander mutations in phage genes by placing them on helper plasmids (HP) and accessory plasmids (AP), respectively (Fig. 1A). These plasmids are continuously replenished within fresh uninfected cells (Fig. 1B). The resulting phagemid evolution system, which we call phagemid-assisted continuous evolution (PACEmid), is based on conditional M13 bacteriophage replication. The selection process takes place inside *E. coli* cells by linking the evolving TF activity to restoring essential phage Gene VI expression (deleted from the HP). In this way, a TF with novel properties can be selected after several cycles of reinfection (Fig. 1B), and the process can be automated (Fig. 1, C and D).

For continuous selection, we found it essential to tune the basal Gene VI expression rate to produce sufficient amounts of phage in the absence of an active TF, reducing the chances of phage loss in the lagoon. We carried out model selections with cI_opt_ [a λ cI optimized mutant with a strong activation region (*18*)] and showed that selection stringency and rate can be tuned by changing the copy number (*19*) of the AP (fig. S2). Furthermore, enrichment of cI_opt_ was more efficient in continuous mode than in batch mode under the same selection pressure (fig. S3), confirming the advantages of continuous selection (*16*, *17*) for accelerated evolution.

We next implemented a mutagenesis device to expand the mutation spectrum beyond combinatorial libraries. On ColE1-derived plasmids, such as our phagemid, the leading-strand replication pol I is gradually replaced by pol III over at least 1.3 kb downstream of the origin of replication (*20*). We therefore characterized the efficiency of three mutagenesis cassettes carrying error-prone pol I [EP pol I (*21*)] or error-prone pol III variants [MP4 and MP6 (*22*)] under the inducible promoters P_BAD_ and P_Llac_ (*23*). Mutation rates were then analyzed by a β-lactamase stop codon reversion assay (fig. S4A), a rifampicin resistance assay (fig. S4, B and C), and by monitoring loss of red fluorescent protein (RFP) function on M13 phagemids after three rounds of batch evolution (fig. S4, D and E). The use of the MP6 (*22*) cassette under the isopropyl β-d-1-thiogalactopyranoside (IPTG)–inducible promoter P_Llac_ (P_Llac_-MP6-SpecR) led to the highest mutation rates, and this was chosen for downstream applications (fig. S4F). When induced with arabinose, the original MP6 leads to a 322,000-fold increase in mutation rate over that of wild-type (WT) *E. coli*. This results in an average of 6.2 × 10^−6^ substitutions per base pair (bp) per generation (*22*).

To validate the use of the adapted MP6 (*22*) mutator cassette for directed evolution, we first evolved an improved orthogonal λ cI TF [cI_4A5T6T,P_; formerly the least active member of our cI toolkit (*12*)] (fig. S5A). The resulting cI variant had a Met-to-Thr mutation at position 42, leading to an improved dual activation-repression of green fluorescent protein (GFP) and mCherry (fig. S5B). The evolved cI_4A5T6T,P (T42)_ up-regulated GFP expression 5.4-fold and led to a 91% repression of mCherry (fig. S5, C and D) (full sequences in the Supplementary Materials). In comparison, expression of the parental cI_4A5T6T,P (M42)_ only displayed a 4.3-fold activation and a 58% repression. Notably, position 42 was not randomized in our previously constructed λ cI library and, thus, confirmed the potential of extra mutagenesis for improved protein activities.

### Directed evolution of Cro activators

**Fig. 2:**
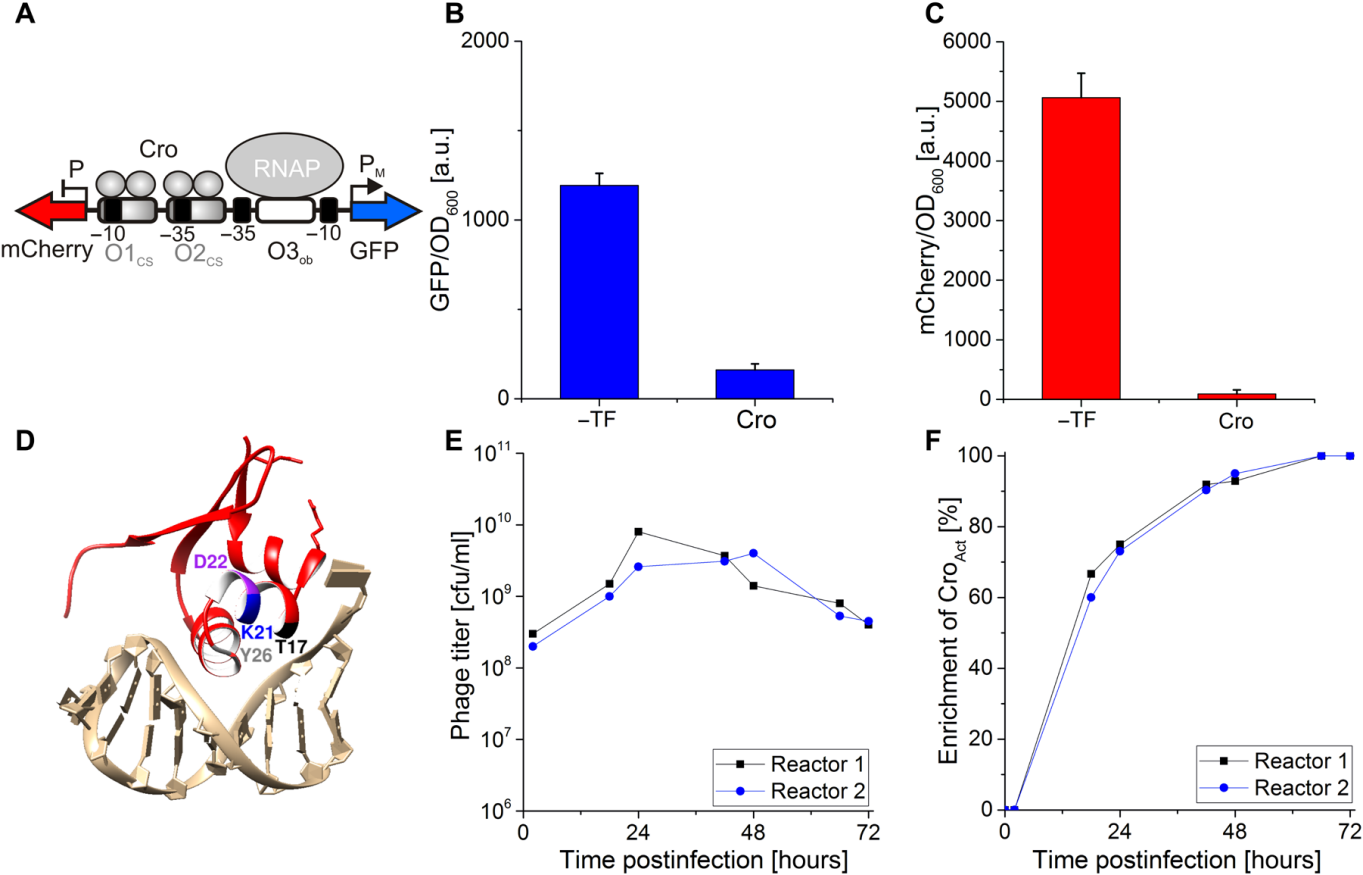
Directed evolution of Cro dual TF activator-repressors. (**A**) Bidirectional promoter designed to activate GFP and repress mCherry. Operators: O1_CS_ and O2_CS_ sites recruit Cro; O3_ob_ is designed to have weaker Cro binding. RNAP, RNA polymerase. (**B**) Despite obliterating the operator O3, expression of Cro still results in partial repression of GFP under the P_M_,_CS_ promoter due to some affinity to the operator 3. (**C**) Cro binds with high affinity to the operators O1_CS_ and O2_CS_ as a dimer, resulting in a strong repression of mCherry. GFP and mCherry expression was normalized to OD_600_ (optical density at 600 nm), and data were obtained from four biological replicates. Error bars show 1 SD. (**D**) Structural model of WT Cro binding DNA, showing key residues (T17, K21, D22, and Y26) randomized in a combinatorial library (Protein Data Bank ID: 6CRO). (**E** and **F**) Time course of the phage titer and enrichment of Cro activators during selection in continuous culture in two bioreactors. Samples were taken from the outflow of each bioreactor, and phage supernatants were analyzed by infecting TG1 cells carrying the reporter plasmid. Cro activators up-regulate GFP and repress mCherry, which can be monitored by plate analysis (enrichment of Cro activators). cfu, colony-forming units. a.u., arbitrary units.

To see whether the optimized phagemid selection system was powerful enough to select a small Cro activator that could function in *E. coli*, we first constructed a bidirectional promoter (P_CS_/P_M,CS_), with three operator sites (O1, O2, O3) (Fig. 2A). O1 and O2 consisted of the λ operator consensus sequence (CS), with the highest reported affinity for λ Cro (*24*, *25*). As Cro binds with a very high affinity to WT O3 (*24*), a mutated O3 site was used to reduce potential autorepression of the P_M,CS_ promoter by λ Cro. Basal promoter activity of P_CS_/P_M,CS_, as well as the effect of Cro on expression levels, was characterized by GFP and mCherry production and showed that WT Cro repressed in both promoter directions (Fig. 2, B and C).

**Fig. 3:**
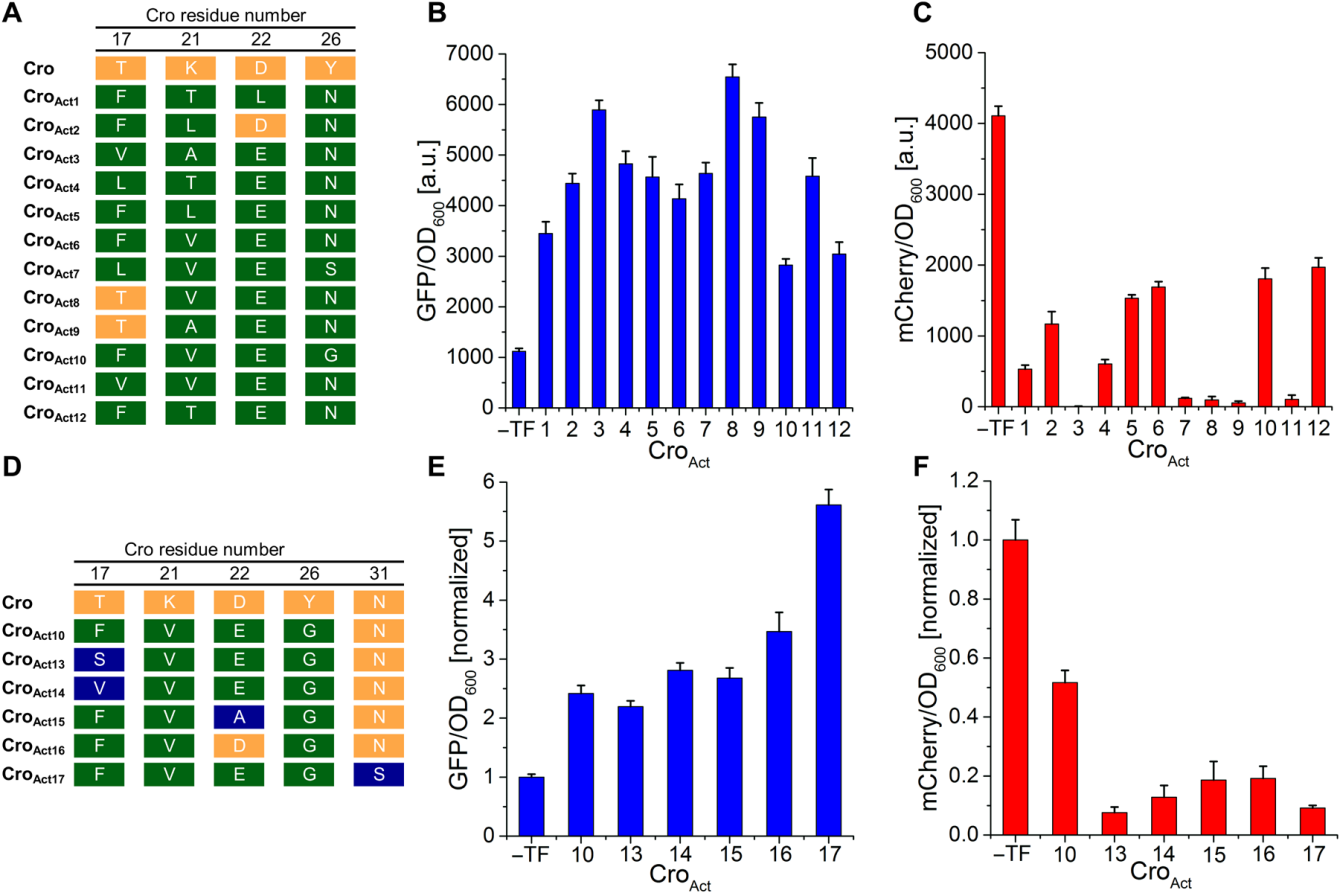
Analysis of Cro dual TF activator-repressors. (**A**) Sequencing results of 12 selected dual Cro activators. WT amino acids are highlighted in orange, and non-WT amino acids in green. (**B** and **C**) Activation and repression of the bidirectional promoter P_CS_/P_M,CS_ by the selected Cro variants. GFP and mCherry expression was normalized to OD_600._ (**D**) Sequencing results from continuous directed evolution using the mutagenesis plasmid on least-active variant Cro_Act10._ (**E** and **F**) Activation and repression of the bidirectional promoter using the variants in (D). All data represent the average of four biological replicates, and error bars correspond to the SD between the measurements.

To search for activators, a combinatorial library of Cro variants was constructed by randomizing four amino acids in α helix two and three (*11*) (Fig. 2D). Three residues (T17, K21, and D22) were in the potential activation patch, whereas the fourth residue (Y26) was upstream of the α helix necessary for DNA binding. We then constructed an AP with Gene VI under the engineered P_M,CS_ promoter, on the pSC101 vector for high selection stringency. The combinatorial Cro library was selected against this AP for 3 days in continuous culture, leading to a reproducible enrichment of Cro activators in two separate bioreactors (Fig. 2, E and F). The selected activators had at least three amino acid substitutions at the randomized positions over WT Cro (Fig. 3A). Notably, 10 of the 12 selected Cro activators contained an asparagine N at position 26. The importance of N26 for activity was confirmed with site-directed mutagenesis (fig. S6, A and B). The activity of the selected Cro activators (Cro_Act_) was then analyzed with the reporter assay (Fig. 2A). Cro_Act_ variants up-regulated GFP expression 2.3- to 5.8-fold and repressed mCherry between 52 and 100% (fig. 3, B and C). In comparison, λ cI expression resulted in a 6.7-fold activation and a 100% repression of the bidirectional P_R_/P_RM_ promoter (fig. S6, C and D). The most active variants possessed the amino acids V17, A21, E22, and N26 (Cro_Act3_), and T17, V21, E22, and N26 (Cro_Act8_) at the randomized positions; both were strong activators and inhibitors of the dual promoter in Fig. 2A.

Next, we explored a wider mutation space using the mutagenesis cassette (P_Llac_-MP6-SpecR). To achieve this, we applied continuous directed evolution for 4 days, using an optimized pLITMUS* vector backbone (fig. S7, A to C) and starting with the least-active variant Cro_Act10_ (Fig. 3A and fig. S7D). Directed evolution was performed under a medium selection pressure (medium copy number plasmid pJPC12) compared to library selections (low copy pSC101; strong selection pressure). This resulted in higher phage production rates and, thus, an increased number of Cro variants. Five additional Cro activators were, thus, evolved with single amino acid changes in the polymerase interaction site (Cro_Act13_ to Cro_Act16_) or DNA binding α helix (Cro_Act17_) (Fig. 3, D to F). Notably, a new amino acid change at position 31, N31S in the DNA binding α helix of Cro_Act17_, had a strong impact on the TF activity. To summarize, we obtained a set of 17 small Cro activators with a broad range of activities.

### Gene network engineering based on Cro activators

**Fig. 4:**
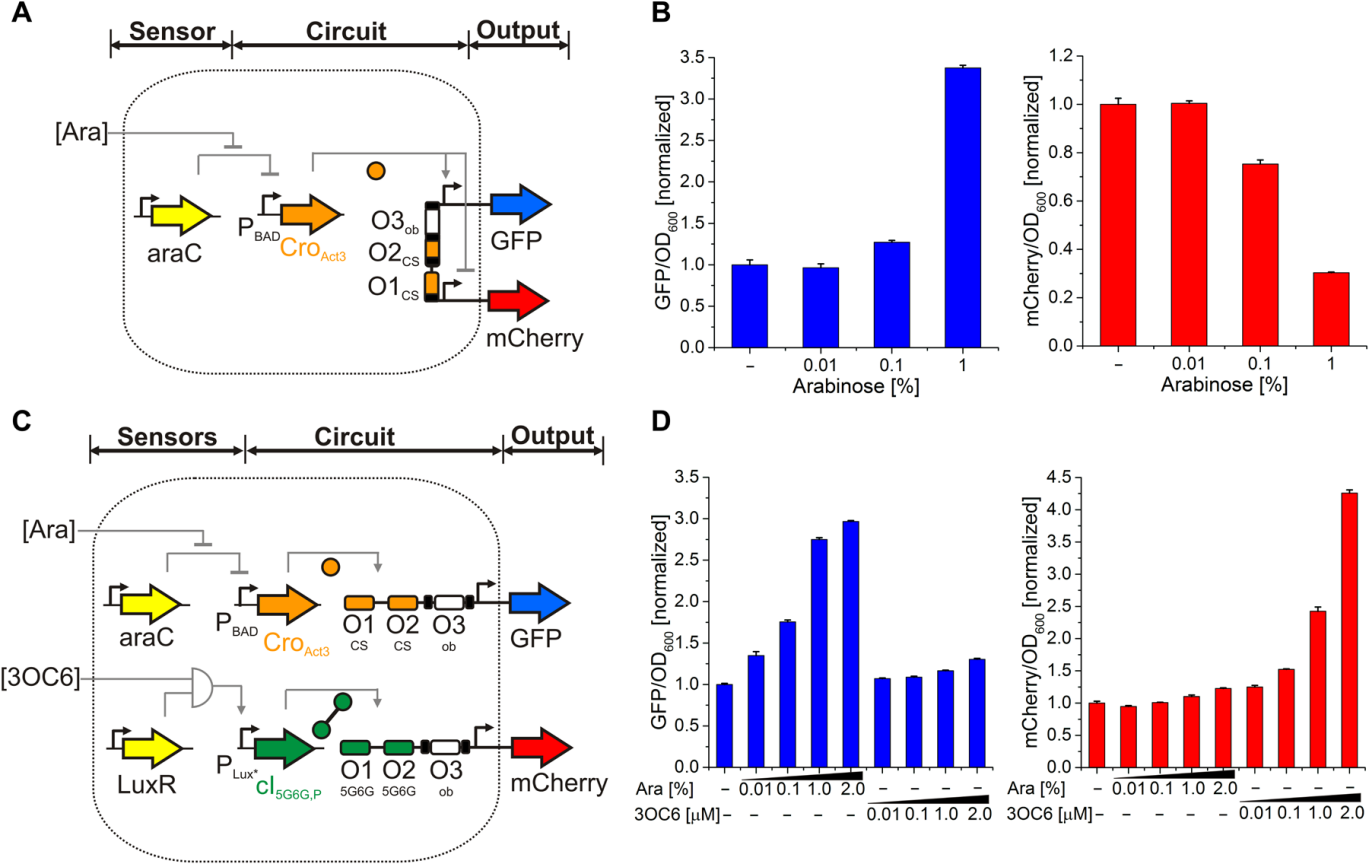
Synthetic gene circuits based on the evolved minimal activator Cro_Act3_. (**A**) Design of a one-input gene network on a bidirectional promoter. The arabinose (Ara)–inducible sensor induces Cro_Act3_ operating on a bidirectional promoter and two reporter genes. Operators: O1_CS_ and O2_CS_ sites (orange) recruit Cro_Act3_; O3_ob_ (white) is an obliterated site to circumvent TF binding. (**B**) Experimental data for the one-input system showing the concentration-dependent response of GFP and mCherry. (**C**) Design of a two-input gene network on two unidirectional promoters. Two sensors (P_BAD_ and P_Lux*_) act on an integrating circuit with two orthogonal TFs (Cro_Act3_ and cI_5G6G,P_) operating on two unidirectional promoters and two reporter genes. Operators: O1_CS_ and O2_CS_ sites (orange) recruit Cro_Act3_; O1_5G6G_ and O2_5G6G_ sites (green) recruit cI_5G6G,P_; O3_ob_ (white) is an obliterated site to circumvent TF binding. (**D**) Experimental data for the two-input system illustrating the concentration-dependent response of GFP and mCherry to the inducers (Ara, 3OC6). All data represent the average of three biological replicates, and error bars correspond to the SD between the measurements.

To test the potential for gene network engineering with the evolved Cro activators, we selected Cro_Act3_ (5.3-fold activation, 100% repression) for use in combination with our set of orthogonal cI variants (*12*). Successful combination would allow the construction of a wide range of gene networks that integrate multiple inputs for activation and/or repression, based on variants of commonly used λ promoters. First, we verified the lack of cross-reactivity of the Cro_Act3_ to any of the synthetic promoters of the orthogonal cI toolkit (fig. S8). Having found no unwanted cross-reactivity, we built two different gene circuits. In the first network, gradual addition of arabinose resulted in expression of Cro_Act3_ and, thus, a concentration-dependent increase in GFP and a decrease in mCherry, as expected (Fig. 4, A and B). Note that arabinose addition generally leads to an induction of more cells rather than increased levels of all cells as the P_BAD_ promoter has an all-or-nothing response (*26*). In the second circuit, expression of the two orthogonal TFs Cro_Act3_ and cI_5G6G,P_, linked to the inducers arabinose and 3OC6-HSL, resulted in a concentration-dependent increase in the reporters GFP and mCherry, as designed (Fig. 4, C and D). Therefore, Cro_Act3_ can be used for gene circuit engineering, either alone or in combination with other orthogonal TFs, and can target unidirectional as well as bidirectional promoters in a concentration-dependent manner.

### Rational engineering of a minimal 63–amino acid dual TF

**Fig. 5:**
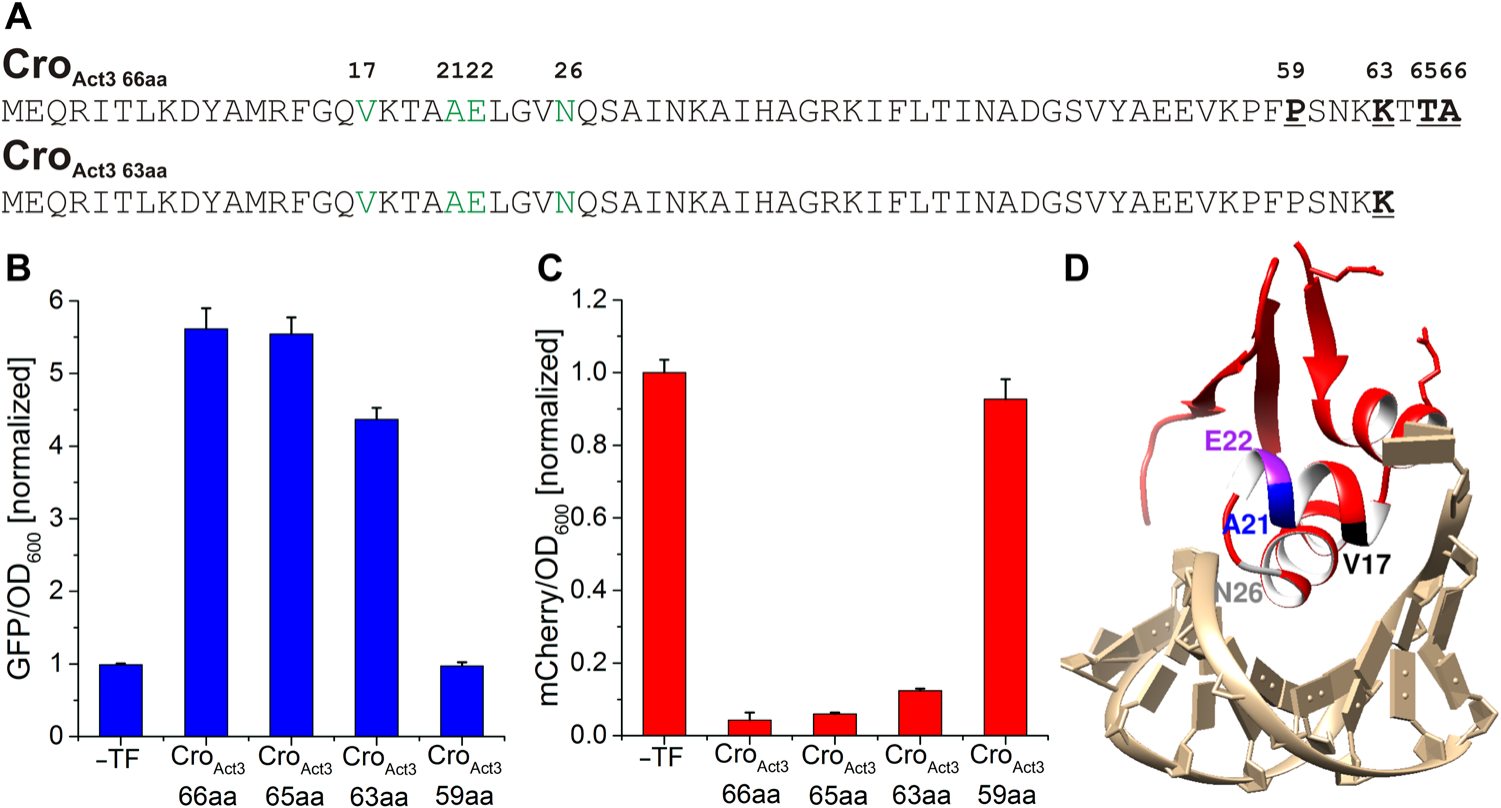
Engineering of a minimal 63–amino acid dual TF. (**A**) Functional breakpoints were identified in the sequence of Cro_Act3_, including a potential structure-breaking Pro^59^, a positive charge patch ending at Lys^63^, and a potentially neutral C-terminal Ala^66^ (bold). The corresponding truncation mutants [59, 63, and 65 amino acids (aa)] were generated; Cro_Act3 63aa_ is shown as an example. Activator mutations to WT λ Cro repressor are highlighted in green. (**B** and **C**) Activation and repression of the bidirectional promoter P_CS_/P_M,CS_ by truncated Cro_Act3_ variants. GFP and mCherry expression were normalized to OD_600_; four biological replicates; error bars represent SD between the measurements. Activation and repression were normalized to the basal expression of each promoter in the absence of any TF on the PM. (**D**) Model indicating key features of the minimal dual TF, Cro_Act3 63aa_.

At 66 amino acids, Cro_Act3_ already had a claim to being the smallest activator and dual TF that enables polymerase recruitment. However, we sought to push the boundaries of the smallest possible such TF. By identifying functional breakpoints in the expected structure (Protein Data Bank ID: 6CRO), we made targeted deletions to the C-terminal end of the TF and analyzed the variants using our reporter assay (Fig. 5A). Thus, we found that a minimal 63–amino acid protein (Cro_Act3 63aa_) is still capable of transcriptional activation or repression in *E. coli* (4.4-fold activation, 88% repression) (Fig. 5, B and C). To our knowledge, this makes it the smallest dual TF that enables polymerase recruitment.

## DISCUSSION

We chose to evolve λ Cro because of its small size, its biological function as counterpart to λ cI, and its use in numerous synthetic biology projects (*27*–*30*). Furthermore, Cro’s α helix two is positioned to be able to interact with the same part of RNA polymerase that λ cI binds. The evolved Cro activators described in this study can easily be constructed by site-directed mutagenesis of WT λ Cro, providing a straightforward approach for users to implement these Cro variants into their synthetic biology projects (components are provided in figs. S9 to S11 and tables S2 to S6).

One basic scientific question that we address in this study is whether a small protein repressor can be converted into a transcriptional activator in vivo. Overall, we conclude that not only is this possible but also that even 63 amino acids are sufficient to carry out dual intracellular transcriptional repression and activation by polymerase recruitment (~4.4-fold). It should be noted that TFs are distinct from DNA binding domains (DBDs). For example, certain zinc fingers can form functional DBDs, such as the 63–amino acid domain from GAGA factor [a single zinc-finger unit flanked by two basic rich regions (*31*)]. However, these interactions are not capable of transcription activation, and the full-length GAGA factor TF is 519 amino acids. A potential alternative to our engineered dual TF is the Arc protein (53 amino acids) from bacteriophage P22, which can act as a repressor or activator (~2.8-fold) (*32*). However, activation does not occur here by recruiting a polymerase but via accelerating the rate at which the polymerase clears the promoter.

In this study, we used a powerful combination of rational design and directed evolution to obtain a set of Cro activators. An alternative strategy is to simply use directed evolution in the absence of a starting library. This would be very interesting as such an approach would lead to detailed information on specific evolution trajectories. In addition, this might give a time frame on how long it would take to walk through sequence space to obtain these beneficial mutations. Last, it would be interesting to further investigate whether our developed directed evolution platform can be easily transferred to evolve other small repressor peptides.

The small size of TFs raises interesting questions for the de novo evolution of DNA binding and polymerase-recruiting proteins from inert protein scaffolds. Cro belongs to the helix-turn-helix (HTH) superfamily (*33*), and this study implies that a relatively small amount of secondary structure, including three short α helices and three β strands, is sufficient to make a compact scaffold that could support minimal gene activation. As long as there are many ways to reach similar folds, the subsequent number of mutations to make a TF may be more tractable for natural selection [~11 mutations for DNA binding (*34*) and ~2 to 5 more for transcription activation]. We note that in yeast, a nine–amino acid peptide is sufficient for transactivation (*35*). Overall, this implies that short peptide scaffolds may have a greater capacity to evolve into DNA binding and RNA polymerase–recruiting proteins than previously thought.

## MATERIALS AND METHODS

### Strains and media

Standard DNA cloning was performed with chemically competent TOP10 cells (Invitrogen) and electrocompetent TG1 cells. Combinatorial library cloning was performed with NEB 5-alpha electrocompetent cells. Phage production was carried out with TOP10, S1030 (*36*), TG1, or BL21(DE3) cells. All phage-assisted infection assays and reporter assays were performed with TG1 cells. Genotypes of all strains are listed in table S2. Cells were grown in 2× TY medium [NaCl (5 g liter^−1^), yeast extract (10 g liter^−1^), tryptone (16 g liter^−1^)], M9 minimal medium [Na_2_HPO_4_ (6.8 g liter−1), KH_2_PO_4_ (3.0 g liter^−1^), NaCl (0.5 g liter^−1^), NH_4_Cl (1.0 g liter^−1^), 2 mM MgSO_4_, 100 μM CaCl_2_, 0.2% (w/v) glucose, 1 mM thiamine-HCl], or Super Optimal broth with Catabolite repression (SOC) medium (Sigma-Aldrich). Chloramphenicol (10 to 25 μg ml^−1^), kanamycin (25 to 50 μg ml^−1^), spectinomycin (25 to 50 μg ml^−1^), ampicillin (50 to 100 μg ml^−1^), tetracycline (5 to 10 μg ml^−1^), and carbenicillin (10 μg ml^−1^) were added where appropriate. IPTG, d-glucose, or l-arabinose was added to the media to induce or repress the promoter P_Llac_ (*23*) or P_BAD_.

### Cloning and plasmid construction

Subcloning was carried out using Gibson Assembly (*37*). GFP (GenBank no. KM229386), RFP, and mCherry (UniProt no. X5DSL3) were used as reporters. The λ Cro regulatory protein (UniProt no. P03040) was used as TF scaffold, and the rpoN promoter (P_rpoN_) was used to express the evolving gene on the phagemid. The stronger activator cI_opt_ (*18*) contains three amino acid changes in the λ cI gene (GenBank no. X00166) at positions 35 to 39 (SVADK to LVAYE). The change in copy number of APs (pSC101, pJPC12, and pJPC13) and the Cro_Act3,Y26_ variant were obtained by site-directed mutagenesis. All mutagenesis plasmids [MP4 and MP6 (*22*); EP pol I and WT pol I (*21*)] were obtained from Addgene and recloned into a vector backbone carrying a spectinomycin resistance gene (SpecR) and a cloDF13 origin of replication to make it compatible with the other plasmids of the directed evolution system. The AraC-P_BAD_ cassette on MP6-SpecR was replaced with the IPTG-inducible promoter P_Llac_ (*23*) (P_Llac_-MP6-SpecR). For the construction of gene circuits, a modified version of the P_Lux_ promoter (P_Lux*_) (*14*) was used to reduce basal expression levels. A degradation tag (AANDENYALVA) was fused to cI_5G6G,P_ at the C-terminal site to decrease basal expression in the absence of an inducer. Promoters, ribosomal binding sites, and terminators were ordered as oligonucleotides (Sigma-Aldrich) or were obtained from previous studies (*38*, *39*). The P_M,CS_ promoter contained the mutated O3 sites TATAAATAGTGGTGATA (*40*) or ACAAACTTTCTTGTATA to bypass autorepression at high λ cI concentrations. Plasmids were purified using the QIAprep Spin Miniprep Kit or the HiSpeed Plasmid Maxi Kit (QIAGEN). Nucleotide sequences of all cloned constructs were confirmed by DNA sequencing (Eurofins Genomics). The DNA sequences of the synthetic promoters are listed in fig. S9. All plasmids and selected primer sequences are listed in tables S3 to S5. Maps for each class of plasmid are highlighted in fig. S10.

### Construction of a combinatorial Cro library

A combinatorial Cro library was cloned on the basis of forward and reverse primers carrying NNS codons (where S = G/C) at positions T17, K21, D22, and Y26, as described previously (table S6) (*15*). Primers were fused by polymerase chain reaction (PCR), and fragments were cloned into the linearized pLITMUS-P_rpoN_-Cro-P_BBa_J23106_-gIII vector by Gibson Assembly. Cells were transformed and plated on 24-cm^2^ Nunc BioAssay Dishes (Thermo Fisher Scientific). Transformation efficiency was estimated by colony counting of plated serial dilutions. The next day, colonies were harvested and phagemid DNA was purified. Ten clones were sequenced to confirm diversity of the library (table S6). Molecular graphics of TFs were obtained with UCSF Chimera (*41*).

### Selection phage production

Selection phage production was performed in BL21(DE3) cells carrying HP-ΔPS-ΔgIII-ΔgVI and pJPC13-ΔPS-P_T7_-gVI or TOP10 cells containing HP-ΔPS-ΔgIII. Cells were made electrocompetent, phagemids were transformed, and cells were grown overnight at 30°C, 250 rpm (Stuart Shaking Incubator SI500), in 2× TY medium supplemented with kanamycin (12.5 μg ml^−1^), ampicillin (50 μg ml ^−1^), and chloramphenicol (12.5 μg ml^−1^) where appropriate. For enrichment assays, plasmids carrying cI_opt_ and RFP were mixed in a ratio of 10^−6^ before transformation. IPTG (0.25 mM) was added to the BL21(DE3) culture after phagemid transformation to induce Gene VI expression. Samples were centrifuged for 10 min at 8000*g*, and supernatants were sterile filtered (0.22-μm pore size, Millex-GV). Phage concentration was analyzed by TG1 infection of diluted phage stocks and colony counting on ampicillin plates.

### Phagemid-assisted batch evolution

TG1 or S1030 cells carrying the helper phage HP-ΔPS-ΔgIII-ΔgVI, a Gene VI–based AP, and, optionally, a mutagenesis plasmid were grown on agar plates (M9 or LB) supplemented with appropriate antibiotics. For TG1-based evolution, starter cultures were inoculated in 2× TY with appropriate antibiotics and grown for 5 to 6 hours at 37°C until the OD_600_ (optical density at 600 nm) reached 0.3 to 0.6. For S1030-based evolution, overnight cultures were inoculated from single colonies. The next day, selection cultures were prepared with a 100-fold dilution of the overnight cultures, and cells were grown for 3 to 4 hours at 37°C until the OD_600_ reached 0.3 to 0.6.

Cultures were infected at a desired multiplicity of infection (MOI), and a chemical inducer was added where appropriate. Cell cultures were incubated for 20 hours at 30°C, 250 rpm (Stuart Shaking Incubator SI500). Overnight cultures were centrifuged for 10 min at 8000*g*, and the phage supernatant was used to start a new round of evolution. After each round, phage supernatants were diluted before infecting TG1 cells carrying an appropriate reporter plasmid. Infected cells were selected on ampicillin plates, and single colonies were grown overnight in 2× TY medium supplemented with ampicillin. Phagemid DNA was purified using the QIAprep Spin Miniprep Kit (QIAGEN) and analyzed by sequencing (Eurofins Genomics).

### Phagemid-assisted continuous evolution

Glass bottles, chemostats, and lagoons connected via biocompatible tubing (Cole-Parmer) were autoclaved and cooled down to room temperature. The autoclaved chemostats and lagoons were placed into two incubators (SID60, Stuart) on individual shakers (Topolino Mobil, IKA). Before each experiment, the bioreactor was equilibrated by pumping 2× TY supplemented with the appropriate antibiotics through the system at 20 ml hour^−1^ (Pharmacia Biotech Pump P-1).

S1030 cells carrying the modified helper phage HP-ΔPS-ΔgIII-ΔgVI, an AP, and, optionally, a mutagenesis plasmid were grown on agar plates supplemented with appropriate antibiotics and 1% (w/v) d-glucose. The next day, 10-ml cultures were inoculated from single colonies, grown overnight at 37°C, 250 rpm, and stored at 4°C. Starter cultures were inoculated with a 100-fold dilution of the overnight culture and grown at 37°C, 250 rpm, until the OD_600_ reached 0.3 to 0.6. Chemostats were filled with 25 ml of this starter culture, and cells were grown at 37°C with magnetic stir-bar agitation. The lagoon was filled with 40 ml of the starter culture, and cells were infected at a MOI of 4 and 1 mM IPTG was added where appropriate. The infected cells were grown at 30°C with magnetic stir-bar agitation. The flow rate of 2× TY supplemented with the appropriate antibiotics was set to 20 ml hour^−1^ to provide continuous supply of media. IPTG (10 mM) in sterile water was added to the lagoon at 2 to 3 ml hour^−1^ to obtain a final concentration of 1 mM for induced mutagenesis (Pharmacia Biotech Pump P-1). Samples were taken from the outflow of the lagoon, centrifuged, and supernatants were stored at 4°C. Samples were serial diluted before infecting TG1 cells with an appropriate reporter plasmid. Phage titers were analyzed by selection on ampicillin plates and colony counting. Single colonies were picked and grown overnight in 2× TY [ampicillin (100 μg ml^−1^)]. Phagemid DNA was purified using the QIAprep Spin Miniprep Kit (QIAGEN) and analyzed by sequencing (Eurofins Genomics). The DNA sequences of selected TFs are listed in fig. S11. For the cI_opt_/RFP enrichment assay, TG1 cells were infected with phage dilutions and plated on ampicillin plates. The ratio of white to red colonies was analyzed by colony counting, and white colonies were linked to cI_opt_ infection by colony PCR.

### β-Lactamase reversion assay

TG1 cells were transformed with the reporter plasmid pLA230 and (i) cloDF-P_BAD_-polA-SpecR, (ii) cloDF-P_BAD_-EPpolA-SpecR, (iii) cloDF-P_BAD_-MP4-SpecR, or (iv) cloDF-P_BAD_-MP6-SpecR and streaked out on LB plates supplemented with spectinomycin, kanamycin, and 1% (w/v) d-glucose. The plasmid pLA230 (*21*) carries a β-lactamase gene with the ochre stop codon TAA at amino acid position 26, which is located 230 bp downstream the origin of replication. The next day, single colonies were picked and grown to the mid-log phase in the presence of 1% d-glucose. Cultures were induced with 1% (w/v) arabinose and incubated for 24 hours at 37°C, 250 rpm (Stuart Shaking Incubator SI500). Cells were diluted and plated on LB plates in the presence or absence of ampicillin and 1% d-glucose and incubated overnight at 37°C. The ratio of the number of ampicillin-resistant colonies divided by the total number of colonies on LB plates was calculated.

### Rifampicin resistance assay

TG1 cells were transformed with MP4-SpecR or MP6-SpecR and plated on LB plates with the appropriate antibiotics and 1% (w/v) d-glucose. The next day, single colonies were picked and grown to the mid-log phase in the presence of 1% d-glucose. Next, cultures were induced with 1% (w/v) arabinose and incubated for 24 hours at 37°C, 250 rpm. Cells were diluted and plated on LB plates with 1% d-glucose in the presence or absence of rifampicin and incubated in the dark for 24 hours at 37°C. The ratio of the number of rifampicin-resistant colonies divided by the total number of colonies on LB plates was calculated.

### RFP mutation assay

S1030 cells carrying HP-ΔPS-ΔgIII-ΔgVI, pJPC12-ΔPS-P_M,CS_-RBS_BBa_B0034_-gVI, and a mutagenesis plasmid (MP4-SpecR, MP6-SpecR, P_Llac_-MP6-SpecR) were grown in 2× TY medium supplemented with antibiotics and 1% d-glucose until the OD_600_ reached 0.4 to 0.6. Cells were infected with an RFP-carrying phagemid (pLITMUS-P_BBa_R0010_-RFP-P_BBa_J23106_-gIII) at MOI 1 and cultured in the presence or absence of an inducer (1% arabinose or 1 mM IPTG) for 20 hours at 30°C, 250 rpm. Phage supernatants were harvested the following day, and experiments were performed for three rounds of evolution in batch mode. TG1 cells were infected with diluted phage supernatants after each round and streaked out on LB plates supplemented with ampicillin. Plates were incubated overnight at 37°C, and the following day, white and red colonies were counted and the ratio was calculated.

### Reporter assay

TG1 cells were transformed with a pJPC12-derived reporter plasmid and a phagemid and selected overnight on agar plates at 37°C. The next day, single colonies were picked for each biological replicate and grown 3 to 5 hours in 1 ml of 2× TY supplemented with chloramphenicol (10 μg ml^−1^) and carbenicillin (10 μg ml^−1^) at 37°C, 250 rpm. The cultures were diluted to OD_600_ 0.01, and 150 μl was added to each well of a 96-well plate. The absorbance at 600 nm, green fluorescence (excitation: 485 nm, emission: 520 nm), and red fluorescence (excitation: 585 nm, emission: 625 nm) were measured every 10 min in a Tecan Infinite F200 PRO plate reader (37°C, shaking between readings) until the *E. coli* cells reached stationary phase. For data analysis, fluorescence readings in the late-exponential phase (OD_600_ of 0.2 for pLITMUS, OD_600_ of 0.9 for pLITMUS*) were used. Both absorbance and fluorescence were background corrected. The fluorescence was then normalized for the number of cells by dividing by the absorbance. The average of three or four biological replicates and the corresponding SD were calculated for each sample.

### Analysis of gene circuits

Single colonies from TG1 cells carrying a pJPC12-derived reporter plasmid and a p15A-derived TF plasmid were grown for 3 to 4 hours in 2 ml of 2× TY supplemented with chloramphenicol (5 μg/ml) and carbenicillin (5 μg/ml). The cultures were diluted to OD_600_ 0.01 in a total volume of 150 μl in each well of a 96-well plate. Arabinose (0.01, 0.1, 1, and 2%) and *N*-(β-ketocaproyl)-l-homoserine lactone (3OC6-HSL; 0.01, 0.1, 1, and 2 μM) were added where appropriate. The absorbance at 600 nm, green fluorescence (excitation: 485 nm, emission: 520 nm), and red fluorescence (excitation: 585 nm, emission: 625 nm) were measured every 10 min in a Tecan Infinite F200 PRO plate reader (37°C, shaking between readings) until the *E. coli* population reached stationary phase. For data analysis, fluorescence readings in the mid- to late-exponential phase were used. Both absorbance and fluorescence were background corrected. The fluorescence was then normalized for the number of cells by dividing by the absorbance. The average of three biological replicates and the corresponding SD were calculated for each sample.

## Supplementary Material

aba2728_SM.pdf

## SUPPLEMENTARY MATERIALS

Supplementary material for this article is available at http://advances.sciencemag.org/cgi/content/full/6/24/eaba2728/DC1

## Appendix

View/request a protocol for this paper from *Bio-protocol*.
