# Supplementary material for: Accelerated evolution of a minimal 63–amino acid dual transcription factor
Source: Sci Adv. 2020 Jun 10;6(24):eaba2728. doi: 10.1126/sciadv.aba2728 (PMC7286687; doi:10.1126/sciadv.aba2728)
Supplement: aba2728_SM.pdf [file aba2728_SM.pdf]

Science Advances
AAAS

advances.sciencemag.org/cgi/content/full/6/24/eaba2728/DC1

Supplementary Materials for

Accelerated evolution of a minimal 63–amino acid dual transcription factor

Andreas K. Brödel, Rui Rodrigues, Alfonso Jaramillo, Mark Isalan*

*Corresponding author. Email: m.isalan@imperial.ac.uk

Published 10 June 2020, *Sci. Adv.* **6**, eaba2728 (2020)
DOI: 10.1126/sciadv.aba2728

This PDF file includes:

Figs. S1 to S11
Tables S1 to S6

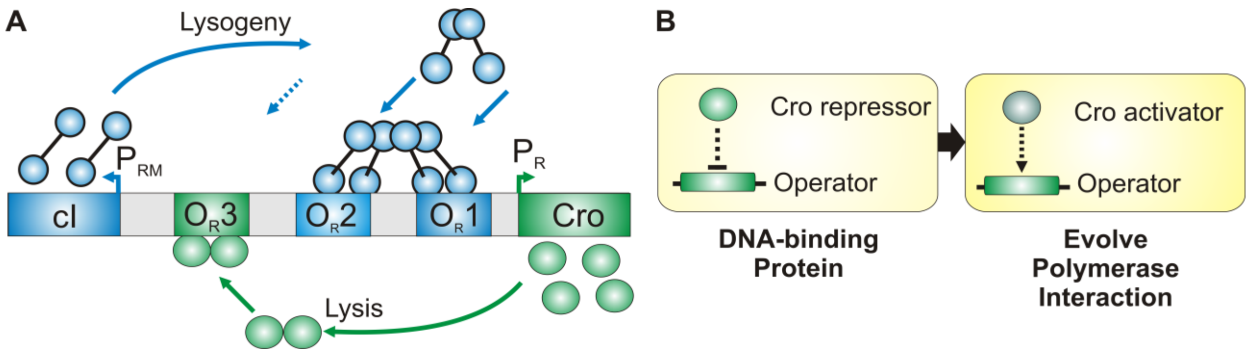

Fig. S1. Directed evolution of small transcriptional activators based on λ Cro for biological computation in living cells. (**A**) Schematic illustration of the phage λ switch, based on dual TF cI and repressor Cro. The O_R region contains three operator sites (O_R1, O_R2, O_R3) and two promoters (P_R and P_{RM}) working in opposite directions. Lambda cI has the highest affinity for O_R1 and O_R2. Binding of cI enables repression of the strong P_R promoter and activation of the weak P_{RM} promoter (lysogenic pathway). A very high cI concentration results in autorepression of P_{RM} by binding to the O_R3 site (dashed line). In contrast, Cro has the highest affinity for O_R3 leading to repression of P_{RM} (lytic pathway). To our knowledge, Cro protein is the smallest transcriptional repressor characterized to date and consists of only 66 amino acids. (**B**) Flow chart of the process to evolve and characterize small transcriptional activators based on λ Cro. There are in total six λ operators (O_L1, O_L2, O_L3, O_R1, O_R2, O_R3) from the leftward P_L and the rightward P_R promoters. Cro has the highest affinity to the consensus sequence (CS) of the six λ operators (*24*). The Cro protein and its consensus operator CS is used as a repressor-operator pair. Cro can potentially be turned into a transcriptional activator by evolving a polymerase interaction site into the repressor molecule. The set of small Cro activators can be used for tunable biological computation, either alone or in combination with other transcription factors.

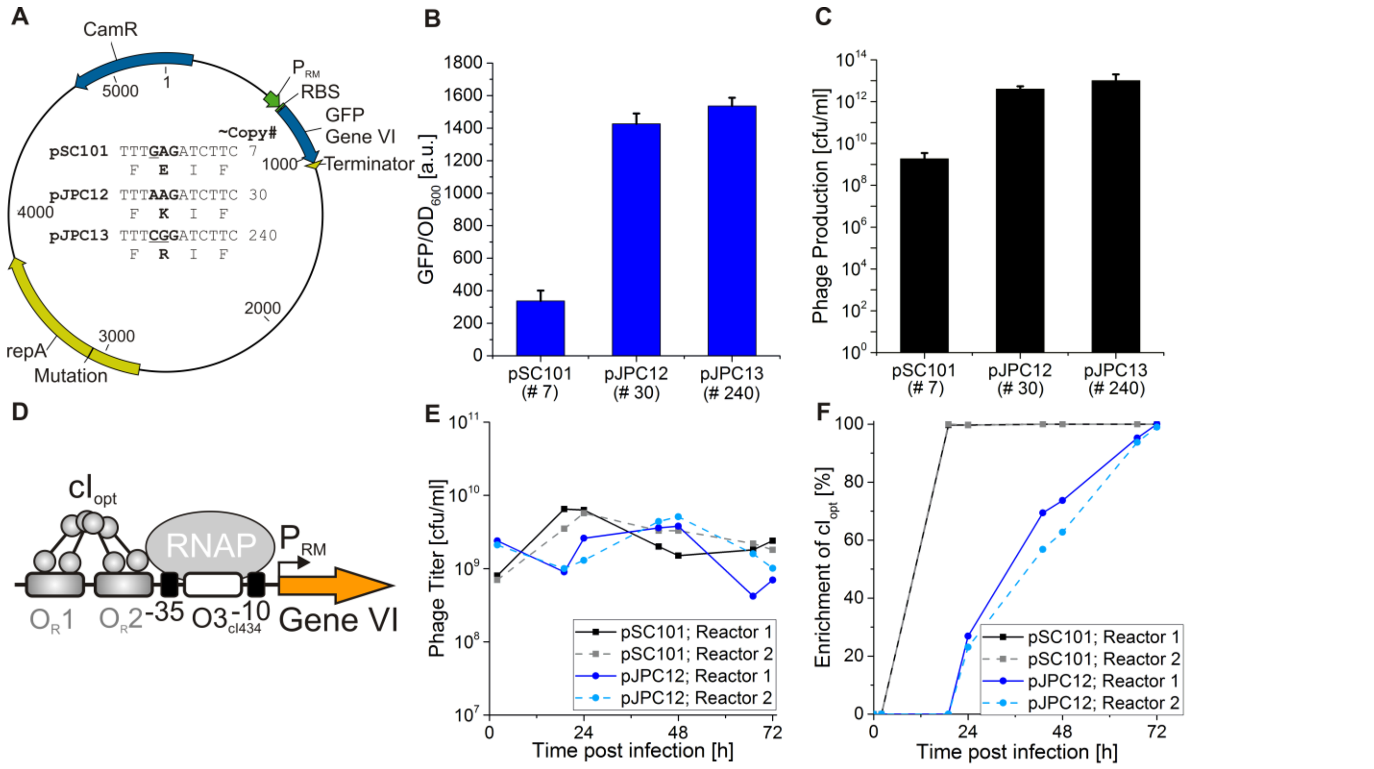

Fig. S2. Copy number adjusts selection stringency in the Phagemid-Assisted Continuous Evolution (PACEmid) system. (**A**) The copy number of the accessory plasmid can be modified by a single amino acid mutation in the repA origin of replication (*19*). The target gene is GFP for reporter assays or Gene VI for selections. (**B**) Effect of the copy number change on basal GFP expression under the synthetic promoter $P_{M,4A5T6T}$ (*12*). Increase of the copy number from 7 (pSC101) to 30 (pJPC12) resulted in a 4.2-fold upregulation of GFP expression in a reporter assay. An additional increase of the copy number to 240 (pJPC13) only marginally increased basal GFP expression. (**C**) Phage production can be tuned by changing the copy number of the accessory plasmid. Phage titers of TG1 cells carrying the helper phage HP-Δg3-Δg6-ΔM13 and the appropriate AP ($P_{M,4A5T6T}$-Gene VI) with three different copy numbers were analyzed. Cells were infected with RFP-expressing phagemid at a MOI of two and phage production was analyzed after 20h post-infection at 30°C in batch mode. Error bars denote the standard deviation of three biological replicates. (**D**) Scheme of the accessory plasmid with Gene VI used for selection. Lambda cI_{opt} (*18*) binds to the promoter and activates Gene VI expression. (**E, F**) Enrichment of cI_{opt} from a mixed phage population with 10^6-fold excess of RFP-expressing phagemid in continuous culture. Selections were performed under two different selection pressures by having the Gene VI circuit on a low copy (pSC101, strong pressure) or a medium copy number plasmid (pJPC12, medium pressure). For each selection stringency, two independent bioreactor experiments were performed. Samples were analyzed twice a day from the

outflow of each lagoon. Enrichment of cI_{opt} was analyzed by calculating the ratio of white (cI_{opt}) to red (RFP) colonies on agar plates.

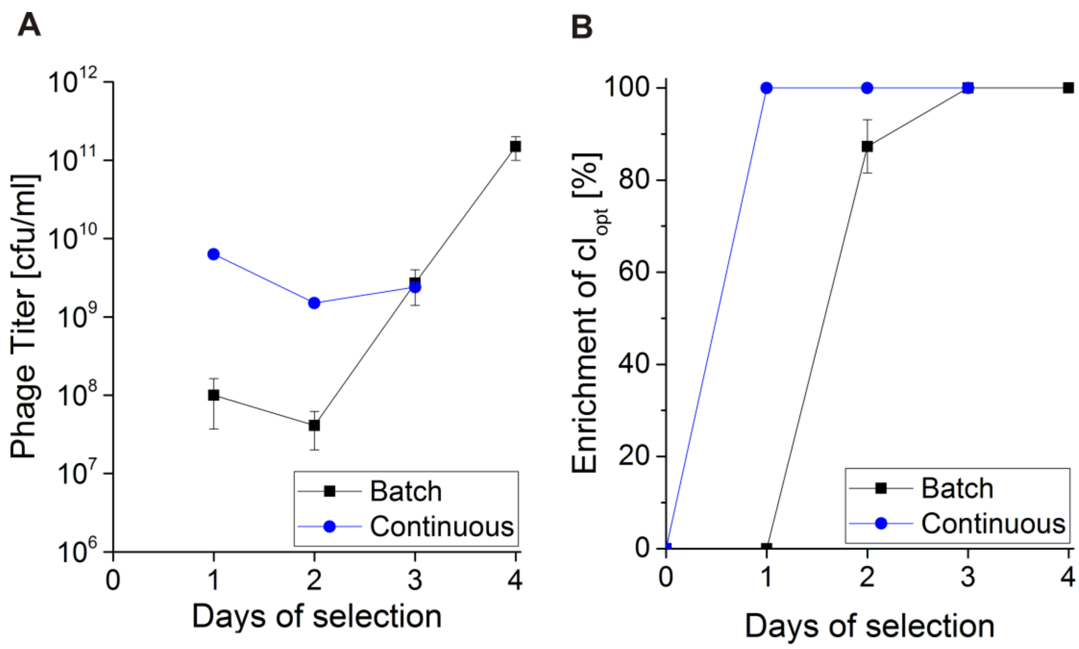

Fig. S3. Enrichment assays of cI$_{opt}$ from mixed phage populations with 10^6-fold excess of RFP-expressing phagemid in batch and continuous mode. Selections were performed under the same selection pressure by having the Gene VI circuit on the low copy number plasmid pSC101 in S1030 cells. Batch cultures were infected at a multiplicity of infection of 0.1 (Round 1) and a 100-fold ratio of supernatant to cell culture was used for consecutive rounds (Round 2 to 4). (**A**) Phage titer in batch and continuous mode. For batch selections, samples were analyzed after each round. (**B**) Enrichment of cI$_{opt}$ was analyzed by calculating the ratio of white (cI$_{opt}$) to red (RFP) colonies on agar plates. In all experiments, phage encoding cI$_{opt}$ were fully enriched after the selection process. Batch data represent the average of three biological replicates and error bars correspond to the standard deviation between the measurements. Continuous data show results of bioreactor 1 (see **Fig. S2 E,F**).

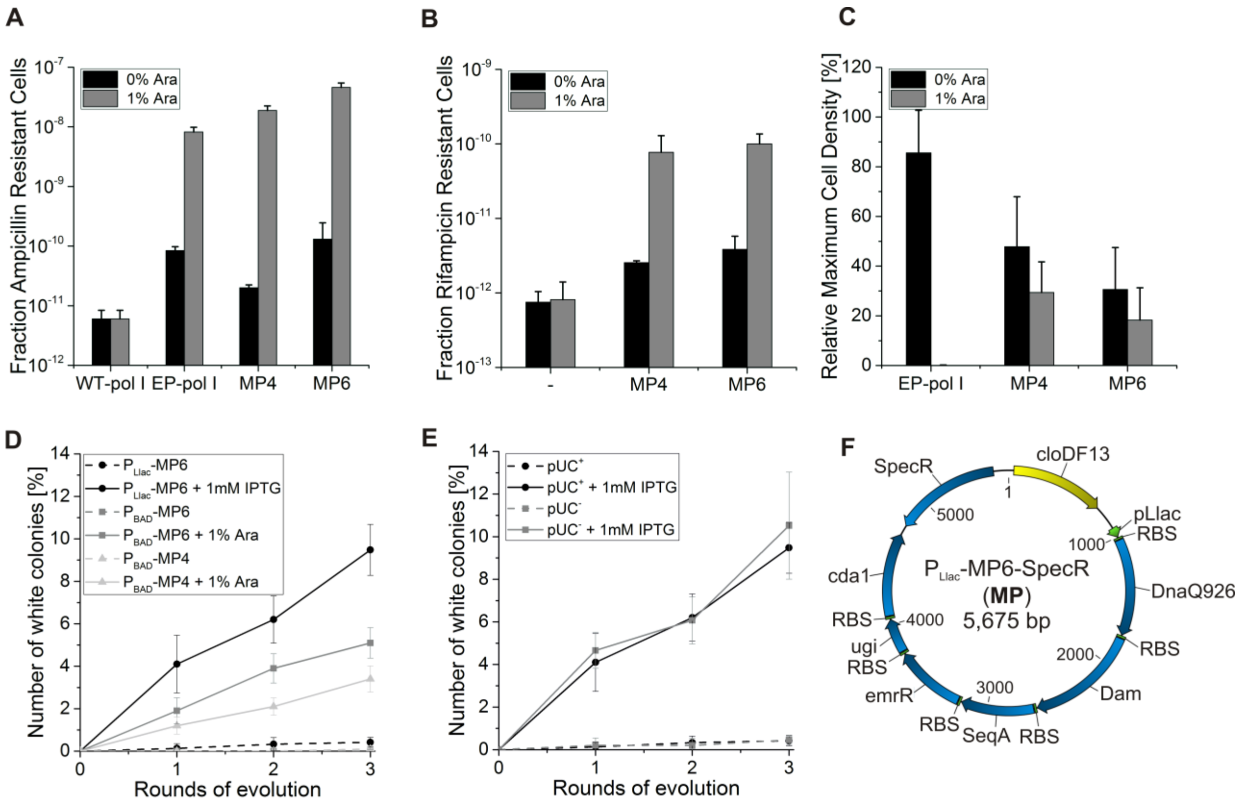

Fig. S4. Implementation of a mutagenesis cassette into the phagemid-assisted directed evolution system. (**A**) Ampicillin reversion assay of error-prone EP-pol I, MP4, and MP6 under an arabinose-inducible promoter P_{BAD} in TG1 cells. Reversion of the stop codon TAA at position 26 of the β-lactamase gene, located 230 bp downstream of the origin of replication, led to ampicillin resistance of the individual cell. WT-pol I under the P_{BAD} promoter was used as a control. Mutation rates were analyzed in the presence or absence of 1% arabinose (Ara). (**B**) Rifampicin resistance assay of TG1 cells carrying MP4 or MP6 in the presence or absence of 1% arabinose. (**C**) Relative maximum cell densities in the presence or absence of 1% arabinose. Cell cultures carrying a mutagenesis plasmid were normalized to a TG1 culture. (**D**) S1030 cells carrying the HP-ΔPS-ΔgIII-ΔgVI, pJPC12-ΔPS-$P_{M,CS}$-RBS_{BBa_B0034}-g6, and a mutagenesis plasmid were infected with an RFP-expressing phagemid and selected for three rounds in the presence or absence of inducer (1% arabinose or 1mM IPTG) in batch mode. Relative mutation rates were analyzed by infecting TG1 cells with diluted phage supernatants after each round of evolution prior to calculating the ratio of white (inactive RFP) to red (active RFP) colonies on ampicillin plates. (**E**) Impact of the pUC orientation on the mutation frequency. Phagemids carrying the pUC in the + or - direction were evolved for three rounds using P_{Llac}-MP6-SpecR and the mutation rates of expressed target protein RFP were analyzed by plate analysis. (**F**) Map of the mutagenesis plasmid with the highest mutation rate.

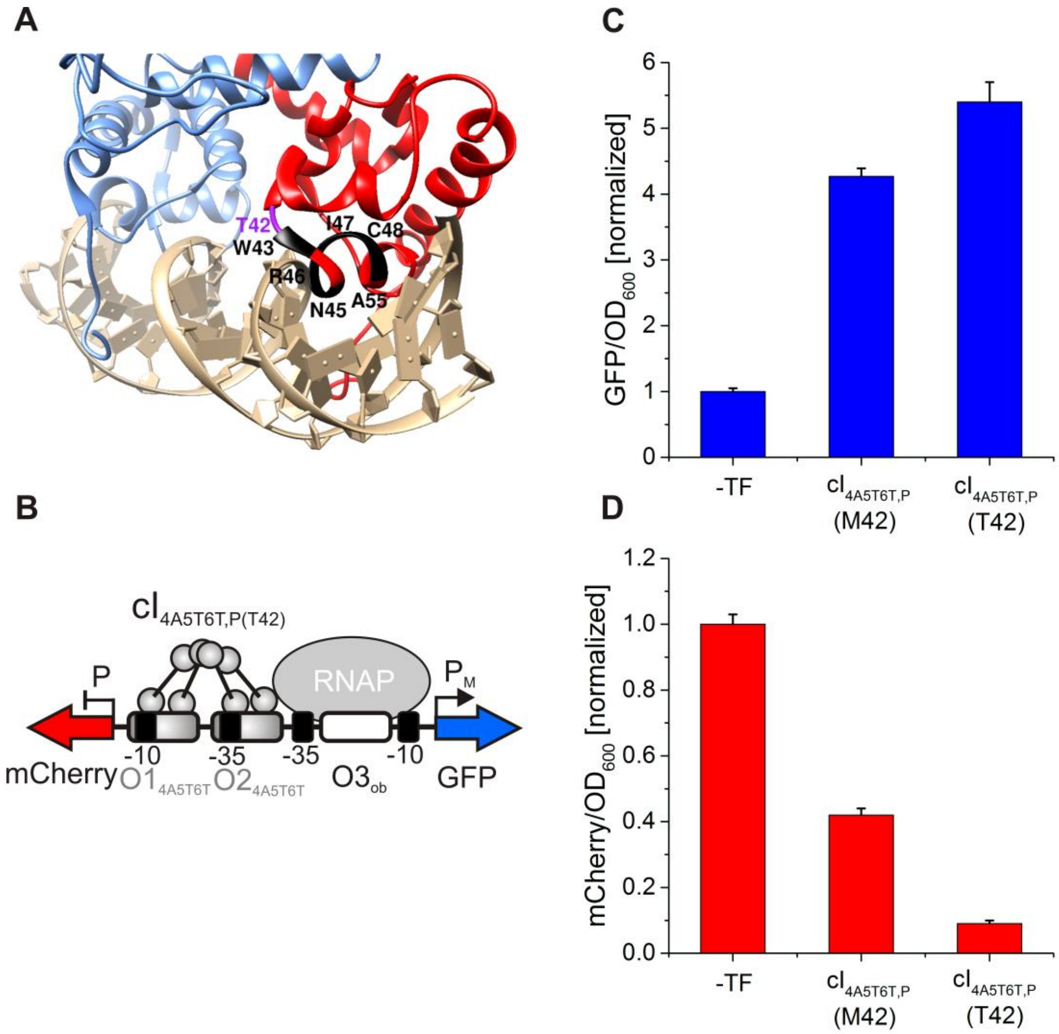

Fig. S5. Directed evolution of an improved orthogonal cI$_{4A5T6T}$ variant using the characterized mutagenesis plasmid. (**A**) The stronger activator of cI$_{4A5T6T,P\ (T42)}$ was evolved after five rounds of batch evolution in TG1 cells carrying the plasmids HP-ΔPS-ΔgIII-ΔgVI, pJPC12-ΔPS-P$_{M,4A5T6T}$-RBS$_{BBa_B0034}$-g6, and P$_{BAD}$-MP6-SpecR. Crystal structure of cI dimer (blue and red) binding to the operator (PDB ID: 3BDN). The amino acid changes in α-helix three of the orthogonal cI$_{4A5T6T,P}$ (*12*) variant are highlighted in black. The evolved amino acid change from M42 to T42 upstream α-helix three is highlighted in purple. (**B**) Scheme of the bidirectional promoter P/P$_{M,4A5T6T}$ used to characterize the evolved cI$_{4A5T6T,P\ (T42)}$ variant with a reporter assay. **(C, D)** The evolved cI$_{4A5T6T,P\ (T42)}$ had a Met to Thr mutation at position 42 leading to an improved DNA binding and thus an increased dual activation/ repression of GFP and mCherry compared to cI$_{4A5T6T,P\ (M42)}$. Error bars denote the standard deviation of three biological replicates. Activation and repression were normalized to the basal expression of each promoter in the absence of a transcription factor (TF) on the phagemid.

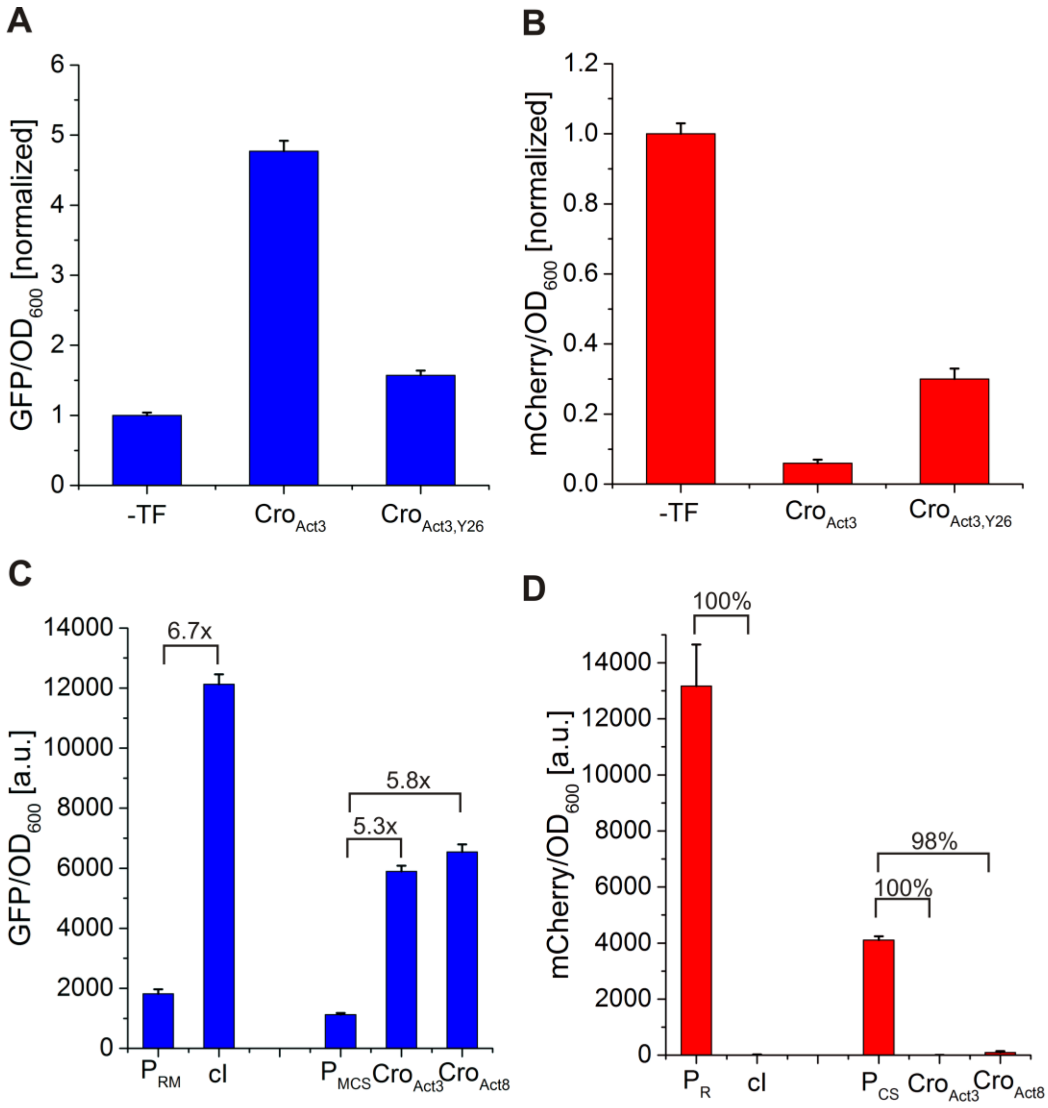

Fig. S6. Characterization of evolved Cro variants. (**A, B**) Importance of asparagine at position 26 for the activity of Cro activators. Dual activation and repression of the bidirectional promoter $P_{CS}/P_{M,CS}$ by $Cro_{Act3,Y26}$ carrying wild-type Y26 was compared to Cro_{Act3} (N26) in a reporter assay. Activation and repression were normalized to the basal expression of each promoter in the absence of a transcription factor (TF) on the phagemid. (**C, D**) Activities of the evolved variants Cro_{Act3} and Cro_{Act8} compared to λ cI. The selected variants Cro_{Act3} and Cro_{Act8} lead to a 5.3-fold or 5.8-fold upregulation of GFP and a 100% or 98% repression of mCherry under the bidirectional promoter $P_{CS}/P_{M,CS}$. In comparison, λ cI expression resulted in a 6.7-fold activation and a full repression of mCherry under the bidirectional promoter P_R/P_{RM}. GFP and mCherry expression was normalized to OD_{600} and data were obtained from four biological replicates. Error bars represent standard deviation between the measurements.

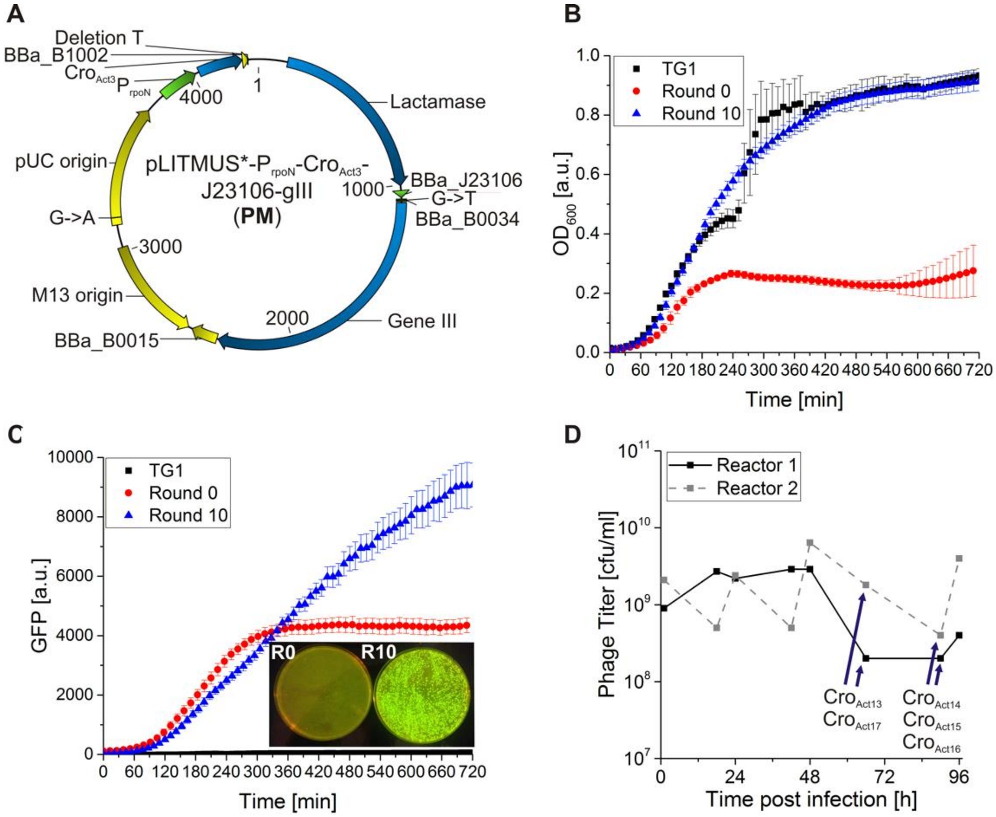

Fig. S7. Characterization of an evolved phagemid backbone carrying Cro$_{Act3}$ and downstream continuous directed evolution starting from the least active variant Cro$_{Act10}$ using this optimized phagemid vector. (**A**) Ten rounds of batch evolution using P$_{Llac}$-MP6-SpecR improved the phagemid backbone carrying Cro$_{Act3,}$ reducing apparent metabolic burden. The evolved phagemid contains a base pair mutation in the origin of replication (G to A), in the ribosomal binding site (RBS) upstream Gene III (G to T) as well as a base pair deletion in the terminator downstream Cro$_{Act3}$ (ΔT). (**B**) Cell growth of TG1 cells carrying the reporter plasmid pJPC12-ΔPS-mCherry-P$_{CS}$/P$_{M,CS}$-GFP and the phagemids before or after ten rounds of batch evolution. Cells carrying the evolved phagemid (Round 10) possess an improved cell growth compared to cells with the parental phagemid (Round 0). (**C**) The improved cell growth affects the overall GFP expression of the reporter plasmid. Bacterial colonies on agar plates depict transformed *E. coli* cells under the UV light (left: Round 0; right: Round 10). Error bars denote the standard deviation of six biological replicates. TG1 cells were used a control. This improved pLITMUS* vector backbone was used in subsequent continuous evolution experiments. (**D**) Continuous directed evolution starting from the least active variant Cro$_{Act10}$. Phage concentrations were measured for two independent bioreactor experiments and Cro variants were obtained at the annotated time points.

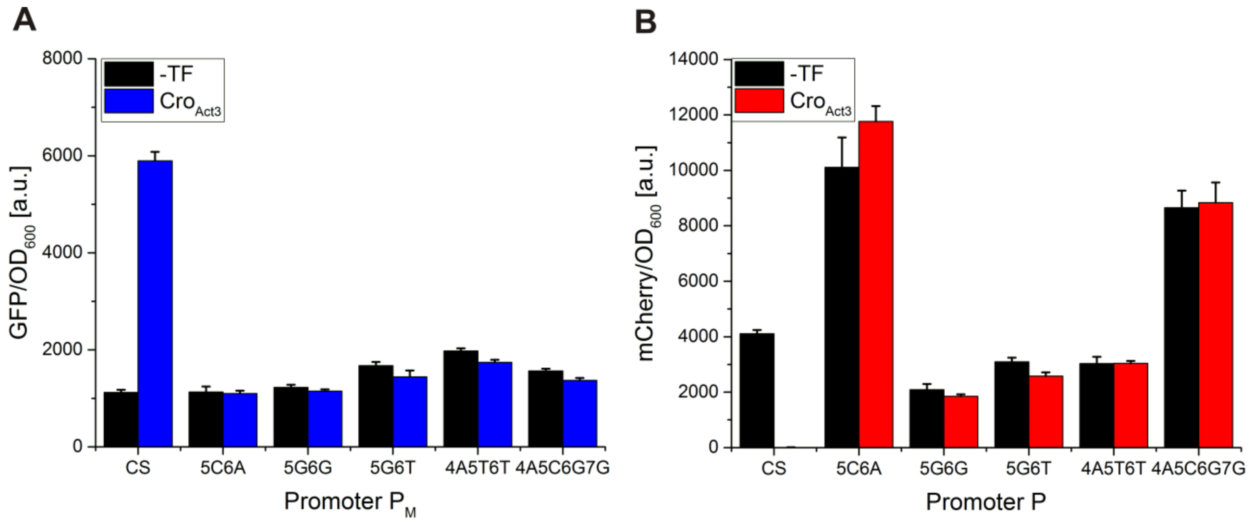

Fig. S8. Cross-reactivity assay: the selected variant Cro_{Act3} does not result in dual activation/ repression of the bidirectional promoters constructed for the orthogonal cI toolkit. (**A**) Basal promoter strengths of the engineered promoters P_M and their activation by Cro_{Act3}. The selected Cro_{Act3} variant upregulates GFP under the consensus promoter $P_{M,CS}$ but not under any other promoter. (**B**) Basal promoter strengths of the engineered promoters P and their repression by Cro_{Act3}. The selected Cro_{Act3} variant represses mCherry under the consensus promoter P_{CS} but not under any other synthetic promoter. GFP and mCherry expression was normalized to OD_{600} and data were obtained from four biological replicates. Error bars represent standard deviation between the measurements.

O1 O2 O3

P_R/P_{RM} (wild-type)

```
GCAACCATTATCACCGCCAGAGGTAAAATAGTCAACACGCACGGTGTTAGATATTTATCCCTTGCGGTGATAGATTTAACGT
CGTTGGTAATAGTGGCGGTCTCCATTTTATCAGTTGTGCGTGCCACAATCTATAAATAGGGAACGCCACTATCTAAATTGCA
```

R1 R2 R3

$P_R/P_{RM\_Reporter}$

```
GCAACCATTATCACCGCCAGAGGTAAAATAGTCAACACGCACGGTGTTAGATATTTATAAATAGTGGTGATAGATTTAACGT
CGTTGGTAATAGTGGCGGTCTCCATTTTATCAGTTGTGCGTGCCACAATCTATAAATATTTATCACCACTATCTAAATTGCA
```

R1 R2 obliterated

$P_R/P_{RM\_Selection}$

```
GCAACCATTATCACCGCCAGAGGTAAAATAGTCAACACGCACGGTGTTAGATATTACAAACTTTCTTGTATAGATTTACAAT
CGTTGGTAATAGTGGCGGTCTCCATTTTATCAGTTGTGCGTGCCACAATCTATAATGTTTGAAAGAACATATCTAAATGTTA
```

R1 R2 OR1 434

$P_{CS}/P_{M,CS\_Reporter}$

```
GCAACCATTATCACCGCCGGTGATAAAATAGTCAACACCGGCGGTGATAGATATTTATAAATAGTGGTGATAGATTTAACGT
CGTTGGTAATAGTGGCGGCCACTATTTTATCAGTTGTGGCCGCCACTATCTATAAATATTTATCACCACTATCTAAATTGCA
```

CS CS obliterated

$P_{CS}/P_{M,CS\_Selection}$

```
GCAACCATTATCACCGCCGGTGATAAAATAGTCAACACCGGCGGTGATAGATATTACAAACTTTCTTGTATAGATTTACAAT
CGTTGGTAATAGTGGCGGCCACTATTTTATCAGTTGTGGCCGCCACTATCTATAATGTTTGAAAGAACATATCTAAATGTTA
```

CS CS OR1 434

Fig. S9. Sequences of synthetic promoters. Synthetic promoters were derived from the natural bidirectional P_R/P_{RM} promoter. Operators are highlighted as follows: O1 blue, O2 green, O3 grey. The natural operator O3 was modified (red) in order to bypass autorepression at high cI concentrations. For cI_{opt} enrichment assays, the OR1 sequence of phage 434 was inserted at position O3. For the evolution of Cro activators, the consensus λ sequence (CS) that is based on the six natural λ operators (O_L1, O_L2, O_L3, O_R1, O_R2, O_R3) from the leftward P_L and the rightward P_R promoters was used at position O1 and O2 because Cro forms the most stable complex with this CS operator. WT cI binding to O3 of the orthogonal promoter $P/P_{M,4A5T6T\_Selection}$ was restored for counterselections by inserting the CS at O3.

O1 O2 O3

P/P$_{M,4A5T6T_Reporter}$

```
GCAACCATTATATTCGCCGAATATAAAATAGTCAAATTCGGCGAATATAGATATTTATAAATAGTGGTGATAGATTTAACGT
CGTTGGTAATATAAGCGGCTTATATTTTATCAGTTTAAGCCGCTTATATCTATAAATATTTATCACCACTATCTAAATTGCA
```

4A5T6T **4A5T6T** **obliterated**

P/P$_{M,4A5T6T_Selection}$

```
GCAACCATTATATTCGCCGAATATAAAATAGTCAAATTCGGCGAATATAGATATTTATCACCGCCGGTGATAGATTTAACGT
CGTTGGTAATATAAGCGGCTTATATTTTATCAGTTTAAGCCGCTTATATCTATAAATAGTGGCGGCCACTATCTAAATTGCA
```

4A5T6T **4A5T6T** **CS**

P/P$_{M,5C6A}$

```
GCAACCATTATCCACGCCGTGGATAAAATAGTCAACCACGGCGTGGATAGATATTTATAAATAGTGGTGATAGATTTAACGT
CGTTGGTAATAGGTGCGGCACCTATTTTATCAGTTGGTGCCGCACCTATCTATAAATATTTATCACCACTATCTAAATTGCA
```

5C6A **5C6A** **obliterated**

P/P$_{M,5G6G}$

```
GCAACCATTATCGGCGCCGCCGATAAAATAGTCAACGGCGGCGCCGATAGATATTTATAAATAGTGGTGATAGATTTAACGT
CGTTGGTAATAGCCGCGGCGGCTATTTTATCAGTTGCCGCCGCGGCTATCTATAAATATTTATCACCACTATCTAAATTGCA
```

5G6G **5G6G** **obliterated**

P/P$_{M,5G6T}$

```
GCAACCATTATCGTCGCCGACGATAAAATAGTCAACGTCGGCGACGATAGATATTTATAAATAGTGGTGATAGATTTAACGT
CGTTGGTAATAGCAGCGGCTGCTATTTTATCAGTTGCAGCCGCTGCTATCTATAAATATTTATCACCACTATCTAAATTGCA
```

5G6T **5G6T** **obliterated**

P/P$_{M,4A5C6G7G}$

```
GCAACCATTATACGGGCCCCGTATAAAATAGTCAAACGGGGCCCGTATAGATATTTATAAATAGTGGTGATAGATTTAACGT
CGTTGGTAATATGCCCGGGCATATTTTATCAGTTTGCCCCGGGCATATCTATAAATATTTATCACCACTATCTAAATTGCA
```

4A5C6G7G **4A5C6G7G** **obliterated**

Fig. S9 (continued). Sequences of synthetic promoters. Synthetic promoters were derived from the natural bidirectional P$_R$/P$_{RM}$ promoter. Operators are highlighted as follows: O1 blue, O2 green, O3 grey. The natural operator O3 was modified (red) in order to bypass autorepression at high cI concentrations. For cI$_{opt}$ enrichment assays, the OR1 sequence of phage 434 was inserted at position O3. For the evolution of Cro activators, the consensus λ sequence (CS) that is based on the six natural λ operators (O$_L$1, O$_L$2, O$_L$3, O$_R$1, O$_R$2, O$_R$3) from the leftward P$_L$ and the rightward P$_R$ promoters was used at position O1 and O2 because Cro forms the most stable complex with this CS operator. WT cI binding to O3 of the orthogonal promoter P/P$_{M,4A5T6T_Selection}$ was restored for counterselections by inserting the CS at O3.

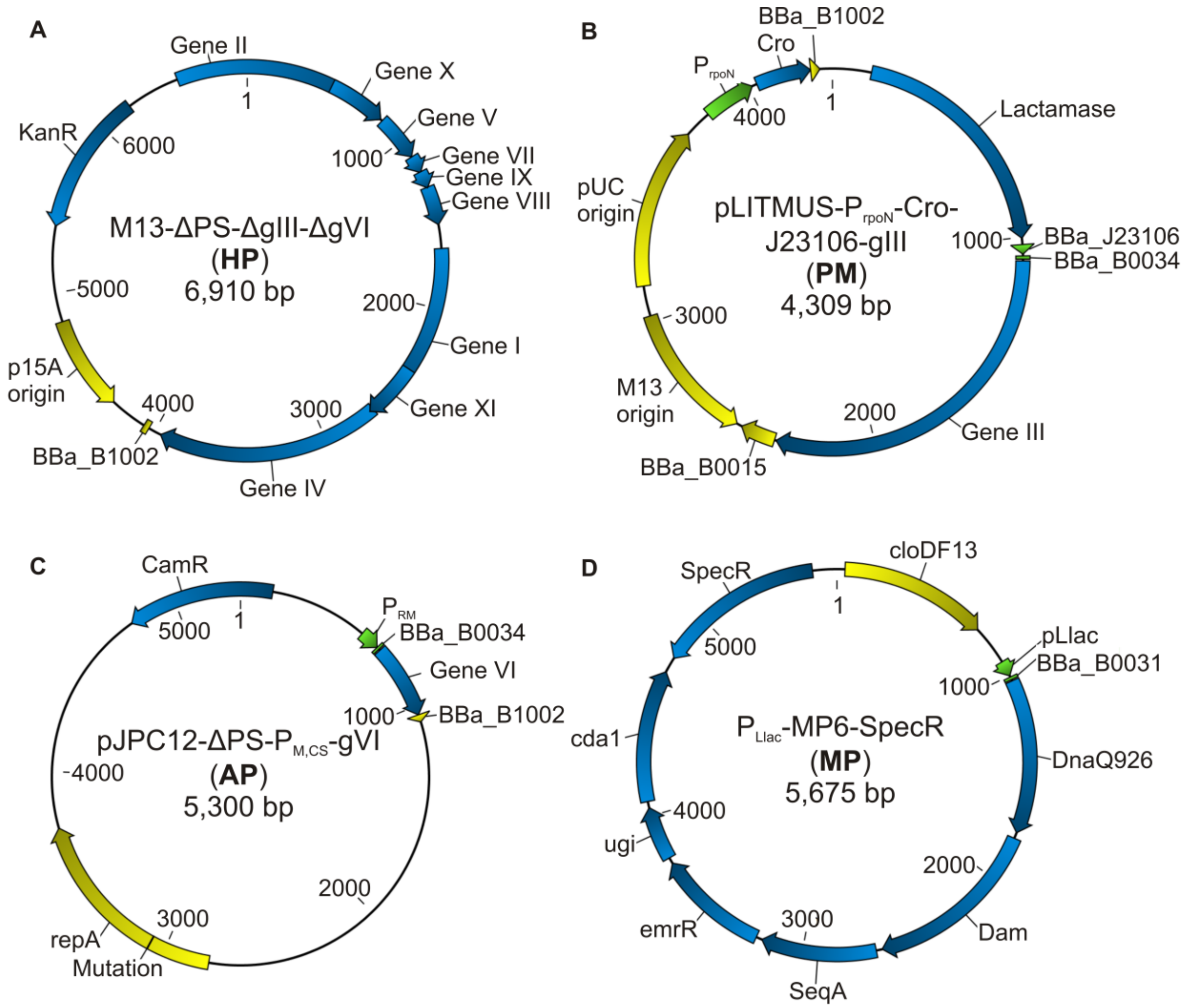

Fig. S10. Maps of plasmids used in the Phagemid-Assisted Continuous Evolution (PACEmid) system. (**A**) The modified helper phage M13KO7-ΔPS-ΔgeneIII-ΔgeneVI (HP) contains all phage genes required for phage replication except the genes III and VI. The weak packaging signal (PS) is removed to bypass helper phage propagation. (**B**) The phagemid pLITMUS-P_{rpoN}-Cro-P_{BBa_J23106}-gIII (PM) provides the evolving gene of interest, the M13 packaging signal (PS) as well as constitutively expressed Gene III. (**C**) The accessory plasmid pJPC12-ΔPS-$P_{M,CS}$-RBS_{BBa_B0034}-gVI (AP) contains a conditional Gene VI expression circuit, activated by an active library member on the phagemid. The copy number of the accessory plasmid can be modified by a single amino acid mutation in the repA origin of replication. (**D**) The mutagenesis plasmid (MP) carries mutator genes (dnaQ926, dam, seqA, emrR, ugi, cda1) under the IPTG-inducible promoter P_{Llac} (*23*).

(A)

Cro

ATGGAACAACGCATAACCCTGAAAGATTATGCAATGCGCTTTGGGCAAACCAAGACAGCTAAAGATCTCGG
CGTATATCAAAGCGCGATCAACAAGGCCATTCATGCAGGCCGAAAGATTTTTTTAACTATAAACGCTGATG
GAAGCGTTTATGCGGAAGAGGTAAAGCCCTTCCCGAGTAACAAAAAAACAACAGCATAA

MEQRITLKDYAMRFGQTKTAKDLGVYQSAINKAIHAGRKIFLTINADGSVYAEEVKPFPSNKKTTA*

(B)

Cro$_{Act1}$

ATGGAACAACGCATAACCCTGAAAGATTATGCAATGCGCTTTGGGCAATTCAAGACAGCTACGCTCCTCGG
CGTAAACCAAAGCGCGATCAACAAGGCCATTCATGCAGGCCGAAAGATTTTTTTAACTATAAACGCTGATG
GAAGCGTTTATGCGGAAGAGGTAAAGCCCTTCCCGAGTAACAAAAAAACAACAGCATAA

MEQRITLKDYAMRFGQFKTATLLGVNQSAINKAIHAGRKIFLTINADGSVYAEEVKPFPSNKKTTA*

Cro$_{Act2}$

GTGGAACAACGCATAACCCTGAAAGATTATGCAATGCGCTTTGGGCAATTCAAGACAGCTCTGGACCTCGG
CGTAAACCAAAGCGCGATCAACAAGGCCATTCATGCAGGCCGAAAGATTTTTTTAACTATAAACGCTGATG
GAAGCGTTTATGCGGAAGAGGTAAAGCCCTTCCCGAGTAACAAAAAAACAACAGCATAA

VEQRITLKDYAMRFGQFKTALDLGVNQSAINKAIHAGRKIFLTINADGSVYAEEVKPFPSNKKTTA*

Cro$_{Act3}$

ATGGAACAACGCATAACCCTGAAAGATTATGCAATGCGCTTTGGGCAAGTGAAGACAGCTGCGGAGCTCGG
CGTAAACCAAAGCGCGATCAACAAGGCCATTCATGCAGGCCGAAAGATTTTTTTAACTATAAACGCTGATG
GAAGCGTTTATGCGGAAGAGGTAAAGCCCTTCCCGAGTAACAAAAAAACAACAGCATAA

MEQRITLKDYAMRFGQVKTAAELGVNQSAINKAIHAGRKIFLTINADGSVYAEEVKPFPSNKKTTA*

Fig. S11. Gene sequences of transcription factors. (**A**) WT Cro (**B**) Selected Cro activator variants**.** Mutations to wild-type λ Cro are highlighted in green. (**C**) Gene sequences of cI variants. Mutations in the DNA-binding site of λ cI to obtain new binding affinities are highlighted in green whereas base pair substitutions to obtain stronger transcriptional activators are highlighted in blue.

Cro$_{\textbf{Act3 59aa}}$

ATGGAACAACGCATAACCCTGAAAGATTATGCAATGCGCTTTGGGCAAGTGAAGACAGCTGCGGAGCTCGG
CGTAAAACCAAAGCGCGATCAACAAGGCCATTCATGCAGGCCGAAAGATTTTTTTAACTATAAACGCTGATG
GAAGCGTTTATGCGGAAGAGGTAAAGCCCTTCCCGTAA

MEQRITLKDYAMRFGQVKTAAELGVNQSAINKAIHAGRKIFLTINADGSVYAEEVKPFP*

Cro$_{\textbf{Act3 63aa}}$

ATGGAACAACGCATAACCCTGAAAGATTATGCAATGCGCTTTGGGCAAGTGAAGACAGCTGCGGAGCTCGG
CGTAAAACCAAAGCGCGATCAACAAGGCCATTCATGCAGGCCGAAAGATTTTTTTAACTATAAACGCTGATG
GAAGCGTTTATGCGGAAGAGGTAAAGCCCTTCCCGAGTAACAAAAAATAA

MEQRITLKDYAMRFGQVKTAAELGVNQSAINKAIHAGRKIFLTINADGSVYAEEVKPFPSNKK*

Cro$_{\textbf{Act3 65aa}}$

ATGGAACAACGCATAACCCTGAAAGATTATGCAATGCGCTTTGGGCAAGTGAAGACAGCTGCGGAGCTCGG
CGTAAAACCAAAGCGCGATCAACAAGGCCATTCATGCAGGCCGAAAGATTTTTTTAACTATAAACGCTGATG
GAAGCGTTTATGCGGAAGAGGTAAAGCCCTTCCCGAGTAACAAAAAAACAACATAA

MEQRITLKDYAMRFGQVKTAAELGVNQSAINKAIHAGRKIFLTINADGSVYAEEVKPFPSNKKTT*

Cro$_{\textbf{Act3,Y26}}$

ATGGAACAACGCATAACCCTGAAAGATTATGCAATGCGCTTTGGGCAAGTGAAGACAGCTGCGGAGCTCGG
CGTATATCAAAGCGCGATCAACAAGGCCATTCATGCAGGCCGAAAGATTTTTTTAACTATAAACGCTGATG
GAAGCGTTTATGCGGAAGAGGTAAAGCCCTTCCCGAGTAACAAAAAAACAACAGCATAA

MEQRITLKDYAMRFGQVKTAAELGVYQSAINKAIHAGRKIFLTINADGSVYAEEVKPFPSNKKTTA*

Fig. S11 (continued). Gene sequences of transcription factors. (**A**) WT Cro (**B**) Selected Cro activator variants**.** Mutations to wild-type λ Cro are highlighted in green. (**C**) Gene sequences of cI variants. Mutations in the DNA-binding site of λ cI to obtain new binding affinities are highlighted in green whereas base pair substitutions to obtain stronger transcriptional activators are highlighted in blue.

Cro$_{Act4}$

ATGGAACAACGCATAACCCTGAAAGATTATGCAATGCGCTTTGGGCAATTGAAGACAGCTACGGAGCTCGG
CGTAAACCAAAGCGCGATCAACAAGGCCATTCATGCAGGCCGAAAGATTTTTTTAACTATAAACGCTGATG
GAAGCGTTTATGCGGAAGAGGTAAAGCCCTTCCCGAGTAACAAAAAAACAACAGCATAA

MEQRITLKDYAMRFGQLKTATELGVNQSAINKAIHAGRKIFLTINADGSVYAEEVKPFPSNKKTTA*

Cro$_{Act5}$

ATGGAACAACGCATAACCCTGAAAGATTATGCAATGCGCTTTGGGCAATTCAAGACAGCTTTGGAGCTCGG
CGTAAACCAAAGCGCGATCAACAAGGCCATTCATGCAGGCCGAAAGATTTTTTTAACTATAAACGCTGATG
GAAGCGTTTATGCGGAAGAGGTAAAGCCCTTCCCGAGTAACAAAAAAACAACAGCATAA

MEQRITLKDYAMRFGQFKTALELGVNQSAINKAIHAGRKIFLTINADGSVYAEEVKPFPSNKKTTA*

Cro$_{Act6}$

ATGGAACAACGCATAACCCTGAAAGATTATGCAATGCGCTTTGGGCAATTCAAGACAGCTGTGGAGCTCGG
CGTAAACCAAAGCGCGATCAACAAGGCCATTCATGCAGGCCGAAAGATTTTTTTAACTATAAACGCTGATG
GAAGCGTTTATGCGGAAGAGGTAAAGCCCTTCCCGAGTAACAAAAAAACAACAGCATAA

MEQRITLKDYAMRFGQFKTAVELGVNQSAINKAIHAGRKIFLTINADGSVYAEEVKPFPSNKKTTA*

Cro$_{Act7}$

ATGGAACAACGCATAACCCTGAAAGATTATGCAATGCGCTTTGGGCAATTGAAGACAGCTGTGGAGCTCGG
CGTAAGCCAAAGCGCGATCAACAAGGCCATTCATGCAGGCCGAAAGATTTTTTTAACTATAAACGCTGATG
GAAGCGTTTATGCGGAAGAGGTAAAGCCCTTCCCGAGTAACAAAAAAACAACAGCATAA

MEQRITLKDYAMRFGQLKTAVELGVSQSAINKAIHAGRKIFLTINADGSVYAEEVKPFPSNKKTTA*

Fig. S11 (continued). Gene sequences of transcription factors. (**A**) WT Cro (**B**) Selected Cro activator variants**.** Mutations to wild-type λ Cro are highlighted in green. (**C**) Gene sequences of cI variants. Mutations in the DNA-binding site of λ cI to obtain new binding affinities are highlighted in green whereas base pair substitutions to obtain stronger transcriptional activators are highlighted in blue.

Cro$_{Act8}$

ATGGAACAACGCATAACCCTGAAAGATTATGCAATGCGCTTTGGGCAAACGAAGACAGCTGTGGAGCTCGG
CGTAAACCAAAGCGCGATCAACAAGGCCATTCATGCAGGCCGAAAGATTTTTTTAACTATAAACGCTGATG
GAAGCGTTTATGCGGAAGAGGTAAAGCCCTTCCCGAGTAACAAAAAAACAACAGCATAA

MEQRITLKDYAMRFGQTKTAVELGVNQSAINKAIHAGRKIFLTINADGSVYAEEVKPFPSNKKTTA*

Cro$_{Act9}$

ATGGAACAACGCATAACCCTGAAAGATTATGCAATGCGCTTTGGGCAAACGAAGACAGCTGCGGAGCTCGG
CGTAAACCAAAGCGCGATCAACAAGGCCATTCATGCAGGCCGAAAGATTTTTTTAACTATAAACGCTGATG
GAAGCGTTTATGCGGAAGAGGTAAAGCCCTTCCCGAGTAACAAAAAAACAACAGCATAA

MEQRITLKDYAMRFGQTKTAAELGVNQSAINKAIHAGRKIFLTINADGSVYAEEVKPFPSNKKTTA*

Cro$_{Act10}$

ATGGAACAACGCATAACCCTGAAAGATTATGCAATGCGCTTTGGGCAATTCAAGACAGCTGTGGAGCTCGG
CGTAGGGCAAAGCGCGATCAACAAGGCCATTCATGCAGGCCGAAAGATTTTTTTAACTATAAACGCTGATG
GAAGCGTTTATGCGGAAGAGGTAAAGCCCTTCCCGAGTAACAAAAAAACAACAGCATAA

MEQRITLKDYAMRFGQFKTAVELGVGQSAINKAIHAGRKIFLTINADGSVYAEEVKPFPSNKKTTA*

Cro$_{Act11}$

ATGGAACAACGCATAACCCTGAAAGATTATGCAATGCGCTTTGGGCAAGTGAAGACAGCTGTGGAGCTCGG
CGTAAACCAAAGCGCGATCAACAAGGCCATTCATGCAGGCCGAAAGATTTTTTTAACTATAAACGCTGATG
GAAGCGTTTATGCGGAAGAGGTAAAGCCCTTCCCGAGTAACAAAAAAACAACAGCATAA

MEQRITLKDYAMRFGQVKTAVELGVNQSAINKAIHAGRKIFLTINADGSVYAEEVKPFPSNKKTTA*

Fig. S11 (continued). Gene sequences of transcription factors. (**A**) WT Cro (**B**) Selected Cro activator variants**.** Mutations to wild-type λ Cro are highlighted in green. (**C**) Gene sequences of cI variants. Mutations in the DNA-binding site of λ cI to obtain new binding affinities are highlighted in green whereas base pair substitutions to obtain stronger transcriptional activators are highlighted in blue.

Cro$_{Act12}$

ATGGAACAACGCATAACCCTGAAAGATTATGCAATGCGCTTTGGGCAATTCAAGACAGCTACCGAGCTCGG
CGTAAACCAAAGCGCGATCAACAAGGCCATTCATGCAGGCCGAAAGATTTTTTTAACTATAAACGCTGATG
GAAGCGTTTATGCGGAAGAGGTAAAGCCCTTCCCGAGTAACAAAAAAACAACAGCATAA

MEQRITLKDYAMRFGQFKTATELGVNQSAINKAIHAGRKIFLTINADGSVYAEEVKPFPSNKKTTA*

Cro$_{Act13}$

ATGGAACAACGCATAACCCTGAAAGATTATGCAATGCGCTTTGGGCAATCCAAGACAGCTGTGGAGCTCGG
CGTAGGGCAAAGCGCGATCAACAAGGCCATTCATGCAGGCCGAAAGATTTTTTTAACTATAAACGCTGATG
GAAGCGTTTATGCGGAAGAGGTAAAGCCCTTCCCGAGTAACAAAAAAACAACAGCATAA

MEQRITLKDYAMRFGQSKTAVELGVGQSAINKAIHAGRKIFLTINADGSVYAEEVKPFPSNKKTTA*

Cro$_{Act14}$

ATGGAACAACGCATAACCCTGAAAGATTATGCAATGCGCTTTGGGCAAGTCAAGACAGCTGTGGAGCTCGG
CGTAGGGCAAAGCGCGATCAACAAGGCCATTCATGCAGGCCGAAAGATTTTTTTAACTATAAACGCTGATG
GAAGCGTTTATGCGGAAGAGGTAAAGCCCTTCCCGAGTAACAAAAAAACAACAGCATAA

MEQRITLKDYAMRFGQVKTAVELGVGQSAINKAIHAGRKIFLTINADGSVYAEEVKPFPSNKKTTA*

Cro$_{Act15}$

ATGGAACAACGCATAACCCTGAAAGATTATGCAATGCGCTTTGGGCAATTCAAGACAGCTGTGGCGCTCGG
CGTAGGGCAAAGCGCGATCAACAAGGCCATTCATGCAGGCCGAAAGATTTTTTTAACTATAAACGCTGATG
GAAGCGTTTATGCGGAAGAGGTAAAGCCCTTCCCGAGTAACAAAAAAACAACAGCATAA

MEQRITLKDYAMRFGQFKTAVALGVGQSAINKAIHAGRKIFLTINADGSVYAEEVKPFPSNKKTTA*

Fig. S11 (continued). Gene sequences of transcription factors. (**A**) WT Cro (**B**) Selected Cro activator variants**.** Mutations to wild-type λ Cro are highlighted in green. (**C**) Gene sequences of cI variants. Mutations in the DNA-binding site of λ cI to obtain new binding affinities are highlighted in green whereas base pair substitutions to obtain stronger transcriptional activators are highlighted in blue.

Cro$_{Act16}$

ATGGAACAACGCATAACCCTGAAAGATTATGCAATGCGCTTTGGGCAATTCAAGACAGCTGTGGATCTCGG
CGTAGGGCAAAGCGCGATCAACAAGGCCATTCATGCAGGCCGAAAGATTTTTTAACTATAAACGCTGATG
GAAGCGTTTATGCGGAAGAGGTAAAGCCCTTCCCGAGTAACAAAAAAACAACAGCATAA

MEQRITLKDYAMRFGQFKTAVDLGVGQSAINKAIHAGRKIFLTINADGSVYAEEVKPFPSNKKTTA*

Cro$_{Act17}$

ATGGAACAACGCATAACCCTGAAAGATTATGCAATGCGCTTTGGGCAATTCAAGACAGCTGTGGAGCTCGG
CGTAGGGCAAAGCGCGATCAGCAAGGCCATTCATGCAGGCCGAAAGATTTTTTAACTATAAACGCTGATG
GAAGCGTTTATGCGGAAGAGGTAAAGCCCTTCCCGAGTAACAAAAAAACAACAGCATAA

MEQRITLKDYAMRFGQFKTAVELGVGQSAISKAIHAGRKIFLTINADGSVYAEEVKPFPSNKKTTA*

Fig. S11 (continued). Gene sequences of transcription factors. (**A**) WT Cro (**B**) Selected Cro activator variants**.** Mutations to wild-type λ Cro are highlighted in green. (**C**) Gene sequences of cI variants. Mutations in the DNA-binding site of λ cI to obtain new binding affinities are highlighted in green whereas base pair substitutions to obtain stronger transcriptional activators are highlighted in blue.

(C)

cI

ATGAGCACAAAAAAGAAACCATTAACACAAGAGCAGCTTGAGGACGCACGTCGCCTTAAAGCAATTTATGA
AAAAAAGAAAAATGAACTTGGCTTATCCCAGGAATCTGTCGCAGACAAGATGGGGATGGGGCAGTCAGGCG
TTGGTGCTTTATTTAATGGCATCAATGCATTAAATGCTTATAACGCCGCATTGCTTGCAAAAATTCTCAAA
GTTAGCGTTGAAGAATTTAGCCCTTCAATCGCCAGAGAAATCTACGAGATGTATGAAGCGGTTAGTATGCA
GCCGTCACTTAGAAGTGAGTATGAGTACCCTGTTTTTTCTCATGTTCAGGCAGGGATGTTCTCACCTGAGC
TTAGAACCTTTACCAAAGGTGATGCGGAGAGATGGGTAAGCACAACCAAAAAAGCCAGTGATTCTGCATTC
TGGCTTGAGGTTGAAGGTAATTCCATGACCGCACCAACAGGCTCCAAGCCAAGCTTTCCTGACGGAATGTT
AATTCTCGTTGACCCTGAGCAGGCTGTTGAGCCAGGTGATTTCTGCATAGCCAGACTTGGGGGTGATGAGT
TTACCTTCAAGAAACTGATCAGGGATAGCGGTCAGGTGTTTTTACAACCACTAAACCCACAGTACCCAATG
ATCCCATGCAATGAGAGTTGTTCCGTTGTGGGGAAAGTTATCGCTAGTCAGTGGCCTGAAGAGACGTTTGG
CTGA

MSTKKKPLTQEQLEDARRLKAIYEKKKNELGLSQESVADKMGMGQSGVGALFNGINALNAYNAALLAKILK
VSVEEFSPSIAREIYEMYEAVSMQPSLRSEYEYPVFSHVQAGMFSPELRTFTKGDAERWVSTTKKASDSAF
WLEVEGNSMTAPTGSKPSFPDGMLILVDPEQAVEPGDFCIARLGGDEFTFKKLIRDSGQVFLQPLNPQYPM
IPCNESCSVVGKVIASQWPEETFG*

cI$_{opt}$

ATGAGCACAAAAAAGAAACCATTAACACAAGAGCAGCTTGAGGACGCACGTCGCCTTAAAGCAATTTATGA
AAAAAAGAAAAATGAACTTGGCTTATCCCAGGAATTGGTCGCATACGAGATGGGGATGGGGCAGTCAGGCG
TTGGTGCTTTATTTAATGGCATCAATGCATTAAATGCTTATAACGCCGCATTGCTTGCAAAAATTCTCAAA
GTTAGCGTTGAAGAATTTAGCCCTTCAATCGCCAGAGAAATCTACGAGATGTATGAAGCGGTTAGTATGCA
GCCGTCACTTAGAAGTGAGTATGAGTACCCTGTTTTTTCTCATGTTCAGGCAGGGATGTTCTCACCTGAGC
TTAGAACCTTTACCAAAGGTGATGCGGAGAGATGGGTAAGCACAACCAAAAAAGCCAGTGATTCTGCATTC
TGGCTTGAGGTTGAAGGTAATTCCATGACCGCACCAACAGGCTCCAAGCCAAGCTTTCCTGACGGAATGTT
AATTCTCGTTGACCCTGAGCAGGCTGTTGAGCCAGGTGATTTCTGCATAGCCAGACTTGGGGGTGATGAGT
TTACCTTCAAGAAACTGATCAGGGATAGCGGTCAGGTGTTTTTACAACCACTAAACCCACAGTACCCAATG
ATCCCATGCAATGAGAGTTGTTCCGTTGTGGGGAAAGTTATCGCTAGTCAGTGGCCTGAAGAGACGTTTGG
CTGA

MSTKKKPLTQEQLEDARRLKAIYEKKKNELGLSQELVAYEMGMGQSGVGALFNGINALNAYNAALLAKILK
VSVEEFSPSIAREIYEMYEAVSMQPSLRSEYEYPVFSHVQAGMFSPELRTFTKGDAERWVSTTKKASDSAF
WLEVEGNSMTAPTGSKPSFPDGMLILVDPEQAVEPGDFCIARLGGDEFTFKKLIRDSGQVFLQPLNPQYPM
IPCNESCSVVGKVIASQWPEETFG*

Fig. S11 (continued). Gene sequences of transcription factors. (**A**) WT Cro (**B**) Selected Cro activator variants**.** Mutations to wild-type λ Cro are highlighted in green. (**C**) Gene sequences of cI variants. Mutations in the DNA-binding site of λ cI to obtain new binding affinities are highlighted in green whereas base pair substitutions to obtain stronger transcriptional activators are highlighted in blue.

cI$_{4A5T6T,P}$ (M42)

ATGAGCACAAAAAAGAAACCATTAACACAAGAGCAGCTTGAGGACGCACGTCGCCTTAAAGCAATTTATGA
AAAAAAGAAAAATGAACTTGGCTTATCCCAGGAATTGGTCGCATACGAGATGGGGATGTGGCAGAACCGCA
TCTGCGCTTTATTTAATGGCATCGCGGCATTAAATGCTTATAACGCCGCATTGCTTGCAAAAATTCTCAAA
GTTAGCGTTGAAGAATTTAGCCCTTCAATCGCCAGAGAAATCTACGAGATGTATGAAGCGGTTAGTATGCA
GCCGTCACTTAGAAGTGAGTATGAGTACCCTGTTTTTTCTCATGTTCAGGCAGGGATGTTCTCACCTGAGC
TTAGAACCTTTACCAAAGGTGATGCGGAGAGATGGGTAAGCACAACCAAAAAAGCCAGTGATTCTGCATTC
TGGCTTGAGGTTGAAGGTAATTCCATGACCGCACCAACAGGCTCCAAGCCAAGCTTTCCTGACGGAATGTT
AATTCTCGTTGACCCTGAGCAGGCTGTTGAGCCAGGTGATTTCTGCATAGCCAGACTTGGGGGTGATGAGT
TTACCTTCAAGAAACTGATCAGGGATAGCGGTCAGGTGTTTTTACAACCACTAAACCCACAGTACCCAATG
ATCCCATGCAATGAGAGTTGTTCCGTTGTGGGGAAAGTTATCGCTAGTCAGTGGCCTGAAGAAACGTTTGG
CTGA

MSTKKKPLTQEQLEDARRLKAIYEKKKNELGLSQELVAYEMGMWQNRICALFNGIAALNAYNAALLAKILK
VSVEEFSPSIAREIYEMYEAVSMQPSLRSEYEYPVFSHVQAGMFSPELRTFTKGDAERWVSTTKKASDSAF
WLEVEGNSMTAPTGSKPSFPDGMLILVDPEQAVEPGDFCIARLGGDEFTFKKLIRDSGQVFLQPLNPQYPM
IPCNESCSVVGKVIASQWPEETFG*

cI$_{4A5T6T,P}$ (T42)

ATGAGCACAAAAAAGAAACCATTAACACAAGAGCAGCTTGAGGACGCACGTCGCCTTAAAGCAATTTATGA
AAAAAAGAAAAATGAACTTGGCTTATCCCAGGAATTGGTCGCATACGAGATGGGGACGTGGCAGAACCGCA
TCTGCGCTTTATTTAATGGCATCGCGGCATTAAATGCTTATAACGCCGCATTGCTTGCAAAAATTCTCAAA
GTTAGCGTTGAAGAATTTAGCCCTTCAATCGCCAGAGAAATCTACGAGATGTATGAAGCGGTTAGTATGCA
GCCGTCACTTAGAAGTGAGTATGAGTACCCTGTTTTTTCTCATGTTCAGGCAGGGATGTTCTCACCTGAGC
TTAGAACCTTTACCAAAGGTGATGCGGAGAGATGGGTAAGCACAACCAAAAAAGCCAGTGATTCTGCATTC
TGGCTTGAGGTTGAAGGTAATTCCATGACCGCACCAACAGGCTCCAAGCCAAGCTTTCCTGACGGAATGTT
AATTCTCGTTGACCCTGAGCAGGCTGTTGAGCCAGGTGATTTCTGCATAGCCAGACTTGGGGGTGATGAGT
TTACCTTCAAGAAACTGATCAGGGATAGCGGTCAGGTGTTTTTACAACCACTAAACCCACAGTACCCAATG
ATCCCATGCAATGAGAGTTGTTCCGTTGTGGGGAAAGTTATCGCTAGTCAGTGGCCTGAAGAAACGTTTGG
CTGA

MSTKKKPLTQEQLEDARRLKAIYEKKKNELGLSQELVAYEMGTWQNRICALFNGIAALNAYNAALLAKILK
VSVEEFSPSIAREIYEMYEAVSMQPSLRSEYEYPVFSHVQAGMFSPELRTFTKGDAERWVSTTKKASDSAF
WLEVEGNSMTAPTGSKPSFPDGMLILVDPEQAVEPGDFCIARLGGDEFTFKKLIRDSGQVFLQPLNPQYPM
IPCNESCSVVGKVIASQWPEETFG*

Fig. S11 (continued). Gene sequences of transcription factors. (**A**) WT Cro (**B**) Selected Cro activator variants**.** Mutations to wild-type λ Cro are highlighted in green. (**C**) Gene sequences of cI variants. Mutations in the DNA-binding site of λ cI to obtain new binding affinities are highlighted in green whereas base pair substitutions to obtain stronger transcriptional activators are highlighted in blue.

Table S1. Size und function of commonly used transcription factors in synthetic biology.

Transcription Factor	Size [amino acids]	Information
**λ** Cro	66	**Repressor** from phage **λ**
P22 Cro	61	**Repressor** from phage P22
**λ** cI	237	**Activator/repressor** from phage **λ**
TetR	208	TetR family **repressor** from *E.coli*
LacI	361	LacI family **repressor** from *E.coli*
LexA	202	LexA family **repressor** from *E.coli*
LuxR	251	LuxR family **activator** from *Vibrio fischeri*
VanR	245	GtbR family **repressor** from *Caulobacter crescentus*
AraC	293	AraC family **activator/repressor** from *E.coli*
PhlF	202	TetR family **repressor** from *Pseudomonas fluorescens*
PcaU	279	IcIR family **repressor** from *Acinetobacter Sp. ADP1*
NahR	302	LysR family **activator** from Pseudomonas putida

**Table S2. Genotypes of *E. coli* strains used in this study. ***The S1030 strain is available on Addgene under the catalog number 105063.

**Strain**	**Genotype**	**Company**
DH5α derivative	fhuA2Δ(argF-lacZ)U169 phoA glnV44 Φ80Δ (lacZ)M15 gyrA96 recA1 relA1 endA1 thi-1 hsdR17	NEB
BL21(DE3)	fhuA2 ompT gal (λ DE3) [dcm] ΔhsdS λ DE3 = λ sBamHIo ΔEcoRI-B int::(lacI::PlacUV5::T7 gene1) i21 Δnin5	NEB
S1030	F’ proA+B+ Δ(lacIZY) zzf::Tn10 lacIQ1 PN25-tetR luxCDE / endA1 recA1 galE15 galK16 nupG rpsL ΔlacIZYA araD139 Δ(ara,leu)7697 mcrA Δ(mrr-hsdRMS-mcrBC) proBA::pir116 araE201 ΔrpoZ Δflu ΔcsgABCDEFG ΔpgaC λ–	Addgene*
TG1	F’[traD36 lacIq Δ(lacZ) M15 proA+B+] glnV (supE) thi-1 Δ(mcrB-hsdSM)5 (rK- mK- McrB-) thi Δ(lac-proAB)	Zymo Research
TOP10	F- mcrA Δ(mrr-hsdRMS-mcrBC) Φ80lacZΔM15 Δ lacX74 recA1 araD139 Δ(araleu)7697 galU galK rpsL (StrR) endA1 nupG	Thermo Fisher Scientific

Table S3. List of plasmids used in this study. The evolved phagemid backbone is annotated with an asterisk. The rotation of the pUC origin of replication is annotated with a minus.

Plasmid	Class	Antibiotic Resistance	Source	Addgene ID
pLITMUS-P_{BBa_J23106}-geneIII	Phagemid	Ampicillin		
pLITMUS-P_{rpoN}-cI-P_{BBa_J23106}-geneIII	Phagemid	Ampicillin		80843
pLITMUS-P_{rpoN}-cI_{opt}-P_{BBa_J23106}-geneIII	Phagemid	Ampicillin		80852
pLITMUS-P_{rpoN}-$cI_{4A5T6T,P\ (M42)}$-P_{BBa_J23106}-geneIII	Phagemid	Ampicillin		80863
pLITMUS-P_{rpoN}-$cI_{4A5T6T,P\ (T42)}$-P_{BBa_J23106}-geneIII	Phagemid	Ampicillin	This work	
pLITMUS-P_{rpoN}-Cro-P_{BBa_J23106}-geneIII	Phagemid	Ampicillin	This work	
pLITMUS-P_{rpoN}-Cro_{Act1}-P_{BBa_J23106}-geneIII	Phagemid	Ampicillin	This work	
pLITMUS-P_{rpoN}-Cro_{Act2}-P_{BBa_J23106}-geneIII	Phagemid	Ampicillin	This work	
pLITMUS-P_{rpoN}-Cro_{Act3}-P_{BBa_J23106}-geneIII	Phagemid	Ampicillin	This work	
pLITMUS-P_{rpoN}-Cro_{Act4}-P_{BBa_J23106}-geneIII	Phagemid	Ampicillin	This work	
pLITMUS-P_{rpoN}-Cro_{Act5}-P_{BBa_J23106}-geneIII	Phagemid	Ampicillin	This work	
pLITMUS-P_{rpoN}-Cro_{Act6}-P_{BBa_J23106}-geneIII	Phagemid	Ampicillin	This work	
pLITMUS-P_{rpoN}-Cro_{Act7}-P_{BBa_J23106}-geneIII	Phagemid	Ampicillin	This work	
pLITMUS-P_{rpoN}-Cro_{Act8}-P_{BBa_J23106}-geneIII	Phagemid	Ampicillin	This work	
pLITMUS-P_{rpoN}-Cro_{Act9}-P_{BBa_J23106}-geneIII	Phagemid	Ampicillin	This work	
pLITMUS-P_{rpoN}-Cro_{Act10}-P_{BBa_J23106}-geneIII	Phagemid	Ampicillin	This work	
pLITMUS-P_{rpoN}-Cro_{Act11}-P_{BBa_J23106}-geneIII	Phagemid	Ampicillin	This work	
pLITMUS-P_{rpoN}-Cro_{Act12}-P_{BBa_J23106}-geneIII	Phagemid	Ampicillin	This work	
pLITMUS-P_{BBa_R0010}-RFP-P_{BBa_J23106}-geneIII	Phagemid	Ampicillin		
pLITMUS*-P_{BBa_J23106}-geneIII	Phagemid	Ampicillin	This work	134352
pLITMUS*-P_{rpoN}-Cro-P_{BBa_J23106}-geneIII	Phagemid	Ampicillin	This work	134353
pLITMUS*-P_{rpoN}-Cro_{Act3}-P_{BBa_J23106}-geneIII	Phagemid	Ampicillin	This work	134354
pLITMUS*-P_{rpoN}-$Cro_{Act3,Y26}$-P_{BBa_J23106}-geneIII	Phagemid	Ampicillin	This work	

pLITMUS*-P$_{rpoN}$-Cro$_{Act3\ 59aa}$-P$_{BBa_J23106}$-geneIII	Phagemid	Ampicillin	This work	
pLITMUS*-P$_{rpoN}$-Cro$_{Act3\ 63aa}$-P$_{BBa_J23106}$-geneIII	Phagemid	Ampicillin	This work	134355
pLITMUS*-P$_{rpoN}$-Cro$_{Act3\ 65aa}$-P$_{BBa_J23106}$-geneIII	Phagemid	Ampicillin	This work	
pLITMUS*-P$_{rpoN}$-Cro$_{Act10}$-P$_{BBa_J23106}$-geneIII	Phagemid	Ampicillin	This work	
pLITMUS*-P$_{rpoN}$-Cro$_{Act13}$-P$_{BBa_J23106}$-geneIII	Phagemid	Ampicillin	This work	
pLITMUS*-P$_{rpoN}$-Cro$_{Act14}$-P$_{BBa_J23106}$-geneIII	Phagemid	Ampicillin	This work	
pLITMUS*-P$_{rpoN}$-Cro$_{Act15}$-P$_{BBa_J23106}$-geneIII	Phagemid	Ampicillin	This work	
pLITMUS*-P$_{rpoN}$-Cro$_{Act16}$-P$_{BBa_J23106}$-geneIII	Phagemid	Ampicillin	This work	
pLITMUS*-P$_{rpoN}$-Cro$_{Act17}$-P$_{BBa_J23106}$-geneIII	Phagemid	Ampicillin	This work	
pLITMUS^{-}-P$_{BBa_R0010}$-RFP-P$_{BBa_J23106}$-geneIII	Phagemid	Ampicillin	This work	
p15A-araC-P$_{BAD}$-Cro$_{Act3}$-P$_{Llac}$-cI$_{5C6A}$-LuxR-P$_{Lux*}$-cI$_{5G6G,P}$LVA	Phagemid	Ampicillin	This work	
M13KO7-ΔPS-ΔgeneIII	Helper phage	Kanamycin	This work	134351
M13KO7-ΔPS-ΔgeneIII-ΔgeneVI	Helper phage	Kanamycin		80840
pSC101-ΔPS-P$_{RM}$-RBS$_{BBa_B0034}$-geneVI	Accessory plasmid	Chloramphenicol	This work	
pSC101-ΔPS-P$_{M,CS}$-RBS$_{BBa_B0034}$-geneVI	Accessory plasmid	Chloramphenicol	This work	134356
pSC101-ΔPS-P$_{M,4A5T6T}$-O3$_{CS}$-RBS$_{BBa_B0034}$-geneVI	Accessory plasmid	Chloramphenicol	This work	
pJPC12-ΔPS-P$_{RM}$-RBS$_{BBa_B0034}$-geneVI	Accessory plasmid	Chloramphenicol		80858
pJPC12-ΔPS-P$_{M,CS}$-RBS$_{BBa_B0034}$-geneVI	Accessory plasmid	Chloramphenicol	This work	
pJPC12-ΔPS-P$_{M,4A5T6T}$-O3$_{CS}$-RBS$_{BBa_B0034}$-geneVI	Accessory plasmid	Chloramphenicol		
pJPC13-ΔPS-P$_{M,4A5T6T}$-O3$_{CS}$-RBS$_{BBa_B0034}$-geneVI	Accessory plasmid	Chloramphenicol	This work	
pJPC13-ΔPS-P$_{T7}$-RBS$_{BBa_B0034}$-geneVI	Accessory plasmid	Chloramphenicol		
pSC101-ΔPS-mCherry-P/P$_{M,4A5T6T}$-GFP	Reporter plasmid	Chloramphenicol	This work	
pJPC12-ΔPS-mCherry-P$_{R}$/P$_{RM}$-GFP	Reporter plasmid	Chloramphenicol		80859
pJPC12-ΔPS-mCherry-P$_{CS}$/P$_{M,CS}$-GFP	Reporter plasmid	Chloramphenicol	This work	134357
pJPC12-ΔPS-mCherry-P/P$_{M,5C6A}$-GFP	Reporter plasmid	Chloramphenicol		80910
pJPC12-ΔPS-mCherry-P/P$_{M,5G6G}$-GFP	Reporter plasmid	Chloramphenicol		80911
pJPC12-ΔPS-mCherry-P/P$_{M,5G6T}$-GFP	Reporter plasmid	Chloramphenicol		80912

pJPC12-ΔPS-mCherry-P/$P_{M,4A5C6G7G}$-GFP	Reporter plasmid	Chloramphenicol		80914
pJPC12-ΔPS-mCherry-P/$P_{M,4A5T6T}$-GFP	Reporter plasmid	Chloramphenicol		80913
pJPC12-ΔPS-$P_{M,5G6G}$-mCherry-$P_{M,CS}$-GFP	Reporter plasmid	Chloramphenicol	This work	
pJPC13-ΔPS-mCherry-P/$P_{M,4A5T6T}$-GFP	Reporter plasmid	Chloramphenicol	This work	
pLA230	Reporter plasmid	Kanamycin		11724
MP4	Mutagenesis plasmid	Chloramphenicol		69652
MP6	Mutagenesis plasmid	Chloramphenicol		69669
cloDF13-P_{BAD}-MP4-SpecR	Mutagenesis plasmid	Spectinomycin	This work	
cloDF13-P_{BAD}-MP6-SpecR	Mutagenesis plasmid	Spectinomycin	This work	
cloDF13-P_{Llac}-MP6-SpecR	Mutagenesis plasmid	Spectinomycin	This work	
cloDF13-P_{BAD}-EP pol I-SpecR	Mutagenesis plasmid	Spectinomycin	This work	
cloDF13-P_{BAD}-WT pol I-SpecR	Mutagenesis plasmid	Spectinomycin	This work	
pEP pol I	Mutagenesis plasmid	Chloramphenicol		11722
pWT pol I	Mutagenesis plasmid	Chloramphenicol		11721

Table S4. Selection of primers used for cloning.

**Name**	**Oligonucleotide sequence**	**Use**
pJPC13-F	5' CCT TTG GTT AAA GGC TTT CGG ATC TTC CAG TGG ACA AAC 3'	Copy number change from pJPC12 to pJPC13
pJPC13-R	5' GTT TGT CCA CTG GAA GAT CCG AAA GCC TTT AAC CAA AGG 3'	Copy number change from pJPC12 to pJPC13
pSC101-F	5' CCT TTG GTT AAA GGC TTT GAG ATC TTC CAG TGG ACA AAC 3'	Copy number change from pJPC12 to pSC101
pSC101-R	5' GTT TGT CCA CTG GAA GAT CTC AAA GCC TTT AAC CAA AGG 3'	Copy number change from pJPC12 to pSC101
$Cro_{Act3,Y26}$-F	5' GCT GCG GAG CTC GGC GTA TAT CAA AGC GCG ATC AAC AAG 3'	Mutagenesis of Cro_{Act3} to $Cro_{Act3,Y26}$
$Cro_{Act3,Y26}$-R	5' CTT GTT GAT CGC GCT TTG ATA TAC GCC GAG CTC CGC AGC 3'	Mutagenesis of Cro_{Act3} to $Cro_{Act3,Y26}$
Library 1-F	5' AG GAG AGT ACG ATT CTG AAC ATG GAA CAA CGC ATA ACC CTG AAA GAT TAT GCA ATG CGC TTT GGG CAA NNS AAG ACA GCT NNS NNS CTC GGC GTA NNS CAA AGC GCG ATC AAC AAG GC 3'	Cloning of the combinatorial library
Library 1-R	5' GCT GTT GTT TTT TTG TTA CTC GGG AAG GGC TTT ACC TCT TCC GCA TAA ACG CTT CCA TCA GCG TTT ATA GTT AAA AAA ATC TTT CGG CCT GCA TGA ATG GCC TTG TTG ATC GCG CTT TG 3'	Cloning of the combinatorial library

Table S5. Sequencing primer used in this study. Five different classes of plasmids are used: Helper phage (HP); Phagemid (PM); Accessory Plasmid (AP); Reporter Plasmid (RP); Mutagenesis Plasmid (MP).

Name	Oligonucleotide sequence	Class
M13KO7-F1	5' GCT ACA ACG GTT AAT TTG C 3'	HP
M13KO7-F2	5' ATG AAA AAG TCT TTA GCC 3'	HP
M13KO7-R1	5' CCA GTT ACA AAA TAA ACA GC 3'	HP
pLITMUS-F1	5' GTC GAT TTT TGT GAT GCT CG 3'	PM
pLITMUS-F2	5' CGT AGT TAT CTA CAC GAC G 3'	PM
pLITMUS-F3	5' AAA AGG ATC TAG GTG AAG 3'	PM
pLITMUS-F4	5' GCC TTT TTA CGG TTC CTG 3'	PM
pLITMUS-F5	5' TTA ATG CGC CGC TAC AGG 3'	PM
pLITMUS-R1	5' GGG TTA TTG TCT CAT GAG CGG ATA C 3'	PM
pLITMUS-R2	5' TGC TTA TAC AAT CTT CCT G 3'	PM
pLITMUS-R3	5' GTC AGT GCG TCC TGC TG 3'	PM
pLITMUS-R4	5' CAC GGA AAT GTT GAA TAC TC 3'	PM
pJPC12-F1	5' AAA CGA CGG CCA GTG AGC 3'	AP + RP
pJPC12-F2	5' AGC CGT ACA TGA ACT GAG 3'	AP + RP
pJPC12-R1	5' GAT AAC AAT TTC ACA CAG G 3'	AP + RP
cloDF13-F1	5' AGA TCA CCA AGG TAG TCG 3'	MP
SeqA-F1	5' CGG AAT TGA TTG AGA AGG 3'	MP
SeqA-F2	5' CGA TTG AAG TTG ATG ATG 3'	MP
pBAD-F1	5' TAT CGC AAC TCT CTA CTG 3'	MP
pBAD-F2	5' CAC TTT GCT ATG CCA TAG C 3'	MP
Dam-F1	5' GGT TGA GCC TTT TGT AGG 3'	MP
Cda1-F1	5' CAC ACC ACC AAA TCT CC 3'	MP
pBAD-R1	5' CAG TAG AGA GTT GCG ATA AAA AG 3'	MP
cloDF13-R1	5' TCG TTT ACA GGG CAA AAG 3'	MP

Table S6. Combinatorial Cro library used in this study. Sequencing results of the combinatorial Cro library. Ten clones were sequenced to confirm diversity. Wild-type amino acids are highlighted in orange, stop codons are annotated in red.

**Library**	**Plasmid**	**Randomized amino acids**	**Size**
1	pLITMUS-P_{rpoN}-Cro-P_{BBa_J23106}-geneIII	T17 K21 D22 Y26	$1.5*10^5$

**Library 1**	**Cro residue number**			
	17	21	22	26
**Cro**	T	K	D	Y
**Clone 1**	I	I	G	Q
**Clone 2**	Y	A	P	L
**Clone 3**	S	V	E	K
**Clone 4**	V	Q	V	G
**Clone 5**	L	G	R	Y
**Clone 6**	I	K	S	M
**Clone 7**	C	A	G	T
**Clone 8**	V	R	W	Y
**Clone 9**	F	*	W	L
**Clone 10**	G	G	V	G
